# Reprogramming the tumor microenvironment by genome editing for precision cancer therapy

**DOI:** 10.1186/s12943-022-01561-5

**Published:** 2022-04-11

**Authors:** Ke Liu, Jia-Jia Cui, Yan Zhan, Qian-Ying Ouyang, Qi-Si Lu, Dong-Hua Yang, Xiang-Ping Li, Ji-Ye Yin

**Affiliations:** 1 Department of Clinical Pharmacology, Xiangya Hospital, Central South University, Changsha, 410078 P. R. China; 2 Institute of Clinical Pharmacology, Central South University, Hunan Key Laboratory of Pharmacogenetics, Changsha, 410078 P. R. China; 3 Engineering Research Center of Applied Technology of Pharmacogenomics, Ministry of Education, 110 Xiangya Road, Changsha, 410078 P. R. China; 4 National Clinical Research Center for Geriatric Disorders, 87 Xiangya Road, Changsha, Hunan 410008 P.R. China; 5 Department of Hematology, Foresea Life Insurance Guangzhou General Hospital, Guangzhou, 510000 P. R. China; 6 Department of Pharmaceutical Sciences, College of Pharmacy and Health Sciences, St. John’s University, 8000 Utopia Parkway, Jamaica, New York, NY 11439 USA; 7 Department of Pharmacy, Xiangya Hospital, Central South University, Changsha, 410008 P. R. China; 8 Hunan Key Laboratory of Precise Diagnosis and Treatment of Gastrointestinal Tumor, Changsha, 410078 P. R. China

**Keywords:** Gene editing, TME, Precision cancer therapy, Reprogramming TME cells, Reprogramming cell-cell communication

## Abstract

The tumor microenvironment (TME) is essential for immune escape by tumor cells. It plays essential roles in tumor development and metastasis. The clinical outcomes of tumors are often closely related to individual differences in the patient TME. Therefore, reprogramming TME cells and their intercellular communication is an attractive and promising strategy for cancer therapy. TME cells consist of immune and nonimmune cells. These cells need to be manipulated precisely and safely to improve cancer therapy. Furthermore, it is encouraging that this field has rapidly developed in recent years with the advent and development of gene editing technologies. In this review, we briefly introduce gene editing technologies and systematically summarize their applications in the TME for precision cancer therapy, including the reprogramming of TME cells and their intercellular communication. TME cell reprogramming can regulate cell differentiation, proliferation, and function. Moreover, reprogramming the intercellular communication of TME cells can optimize immune infiltration and the specific recognition of tumor cells by immune cells. Thus, gene editing will pave the way for further breakthroughs in precision cancer therapy.

## Introduction

**Fig. 1:**
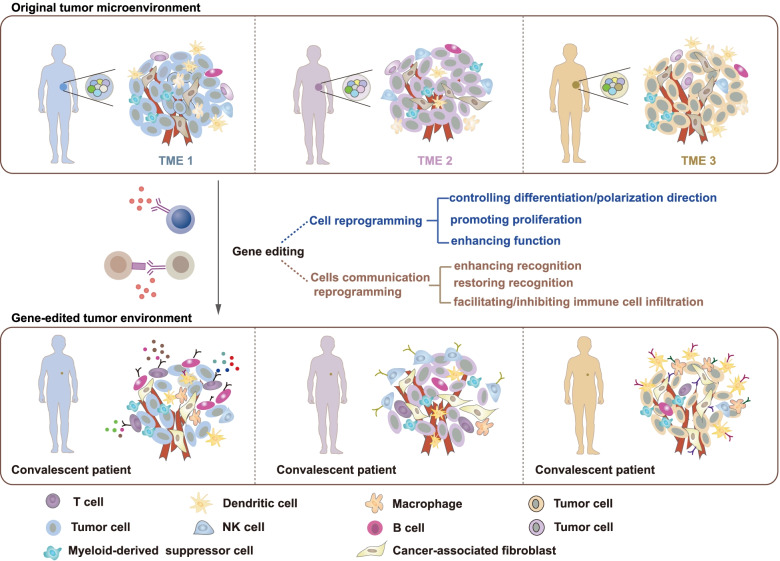
Reprogramming the TME via genome editing in precision cancer therapy. The personalized treatment process based on TME reprogramming is summarized in this figure. The top panel represents different TMEs in different tumor patients, and the middle panel represents gene editing strategies used for TME cell reprogramming and cellular communication reprogramming. The gene-edited TME of convalescent patients is shown in the bottom of the figure

The cellular environment in which tumor cells reside is called the tumor microenvironment (TME). It consists of immune cells, fibroblasts, endothelial cells and mesenchymal cells [1]. The TME allows tumor cells to escape host immunity and is involved in cancer development and metastasis. Recent studies have shown that the TME varies among individuals and is strongly associated with clinical prognosis [2, 3]. Therefore, TME reprogramming is becoming an essential strategy for cancer treatment (Fig. 1).

The understanding of TME reprogramming was previously restricted due to technological limitations. Researches in this field have rapidly increased with recent advances in gene editing technologies. Currently, it is possible to individualize cancer therapy by reprogramming different cells in the TME, and some of these strategies have already been used in the clinic. This personalized approach represents one of the most attractive and promising strategies for cancer therapy in the future. However, systematic reviews on the role of gene editing in TME reprogramming are scarce. Herein, we summarize the recent advances in TME reprogramming based on the application of gene editing to affect TME cells and their communication.

## Gene editing technologies

**Fig. 2:**
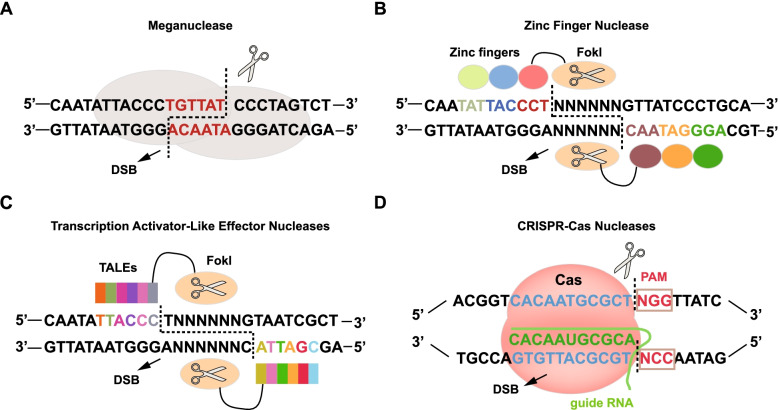
Gene editing technologies. Editing principles of the four technologies. **A** Meganucleases, **B** Zinc Finger Nucleases, **C** Transcription Activator-Like Effector Nucleases, **D** CRISPR-Cas Nucleases. DSB: DNA double-strand break; PAM: protospacer adjacent motif

In 1952, Salvador Luria discovered the DNA restriction-modification system of bacteria. Based on this discovery, researchers have created a series of technologies to modify genes, including gene targeting and RNA interference. After more than half a century of perfecting and improving these approaches, gene editing technologies have become increasingly mature. Currently, there are four main types of gene editing technologies: Meganucleases (MegaNs), Zinc finger nucleases (ZFNs), Transcription activator-like effector nucleases (TALENs), and Clustered regularly interspaced short palindromic repeats (CRISPR)/CRISPR-associated proteins (Cas) systems (Fig. 2, Table 1).

**Table 1:** Comparison of MegaNs, ZFNs, TALENs and CRISPR-Cas systems

	MegaNs	ZFNs	TALENs	CRISPR-Cas systems
**DNA recognition domain**	Homing endonucleases (binding domain)	Zinc finger protein	a series of repeats of transcription activator-like effector proteins	Single-strand guide RNA
**DNA cleavage domain**	Homing endonucleases (cleaving domain)	*FokI* endonuclease	*FokI* endonuclease	Cas protein
**Target sequence size**	14–40 bp	18–36 bp	28–40 bp	20 bp gRNA sequence and PAM sequence
**Mechanism of target specificity**	Naturally occurring	One zinc finger protein recognizes three nucleotides	One repeat of transcription activator-like effector proteins recognizes one nucleotide	sgRNA imparts targeting specificity through DNA-RNA complementarity
**Advantages**	High specificity Easy to deliver in vivo	Moderately specificity Easy to deliver in vivo	High specificity Relatively easy to engineer	High specificity Easy to engineer
**Disadvantages**	Hard to engineer	Hard to engineer	Relatively hard to deliver	Limited in vivo delivery

MegaNs splice DNA at specific recognition sites that naturally and occasionally occur in any genome. MegaNs have often been used to edit genes in crop or animal cells but rarely those in human cells [4]. ZFNs are artificially engineered endonucleases that consist of a DNA recognition domain and the nonspecific endonuclease FokI [5]. The former is responsible for identifying the base sequence of DNA-specific sites, and the latter performs the splicing function. ZFNs have been used to edit tumor and immune cells to optimize precision cancer therapy [6]. Similar to ZFNs, TALENs contain a recognition domain that is composed of highly conserved repeats derived from transcription activator-like effectors (TALEs) [7]. This customizable DNA-binding domain guides the FokI enzyme to trim sequences in the specified site. A repeat can recognize only one nucleotide, which makes the editing performed by TALENs more flexible and specific [8]. T cells engineered with TALENs enhance the antitumor efficacy of adoptive immunotherapy [9]. TALENs have also been applied to edit the genome of human induced pluripotent stem cells (iPSCs), making these cells differentiate into immune cells with potential antitumor activity [10]. The emergence of the CRISPR/Cas system produced a revolution in gene editing technology. CRISPR/Cas is an acquired immune system in bacteria that is used to fight invading DNA, plasmids, and phages [11]. The CRISPR/Cas system consists of CRISPR-derived RNA (crRNA) and Cas proteins. crRNA directs the Cas proteins to a specific location, while the Cas proteins are responsible for splicing DNA [12, 13]. The CRISPR/Cas system, the fastest growing editing technology in recent years, can also be used to eliminate tumors by editing target genes in TME cells. Currently, the leading gene editing technologies used to reprogram the TME are TALENs and the CRISPR/Cas system. The effectiveness and safety of gene editing technologies in cancer treatment have been established in several clinical trials [14, 15].

## Cell reprogramming

**Fig. 3:**
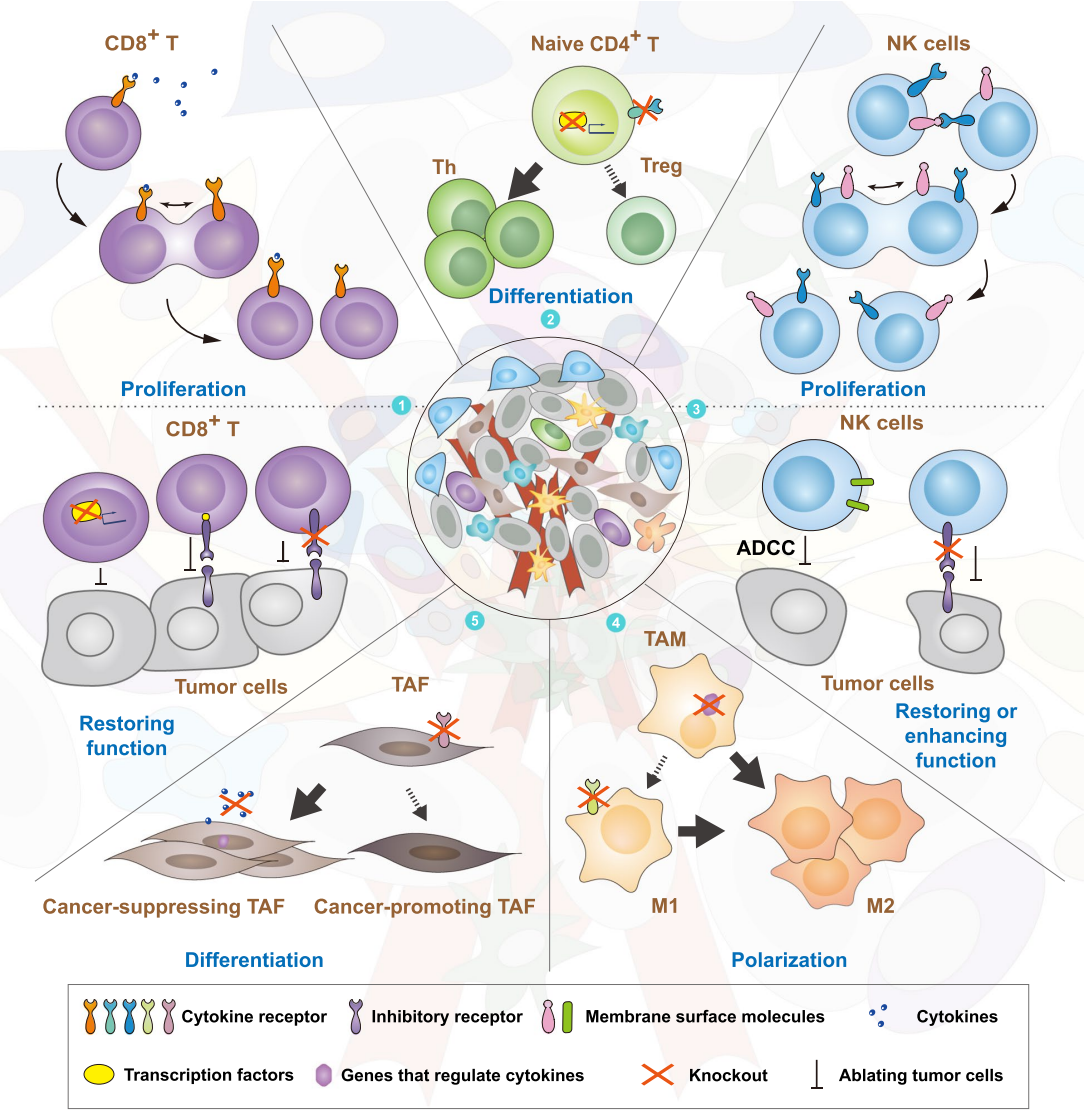
Reprogramming TME cells via gene editing. Gene editing is mainly used to reprogram CD8^+^ T cells, CD4^+^ T cells, NK cells, TAMs, and TAFs. ① The proliferation of CD8^+^ T cells is promoted by editing cytokine receptors on the CD8^+^ T-cell surface (above). The function of exhausted CD8^+^ T cells is restored by knocking out inhibitory receptors, altering the expression of transcription factors, or fusing inhibitory receptors and costimulatory domains (below). ② The differentiation of naive CD4^+^ T cells is regulated by altering the expression of transcription factors or surface-localized cytokine receptors. ③ The proliferation of NK cells is promoted by editing cytokines on the NK-cell surfaces (above). The function of exhausted NK cells is restored by knocking out inhibitory receptors, and their cytotoxicity is enhanced by altering the expression of genes involved in the ADCC process (below). ④ The polarization of M1 macrophages can be promoted by knocking out genes regulating cytokines in M0 macrophages or cytokine receptors expressed on M1 macrophages. ⑤ The differentiation of TAFs is regulated by altering their expression of cytokine receptors, and the function of cancer-promoting TAFs is weakened by inhibiting their release of inflammatory factors

Cells in the TME can be categorized into immune and nonimmune cells. Both are important for the development of tumors and can be reprogrammed. Different gene editing strategies should be selected based on cell function and characteristics. In general, gene editing can be performed to eliminate tumors based on three aspects: controlling the direction of naive cell differentiation/polarization, promoting proliferation, and enhancing the function of effector cells (Fig. 3, Table 2).

**Table 2:** Potential edited genes that regulate cells proliferation, differentiation or function in TME

Cell type	Function	Gene	Reference
**CD8**^**+**^**T cell**	Promote proliferation	IL-2Rα, IL-2Rβ, IL-4Rα, IL-7Rα, IL-10Rα, IL-10Rβ, IL-12R, IL-15Rα, GITR, HDAC1, NFAT1, NR4A1, SLAT, SUMO2, TL1A, DOCK8, TIS21, STAT6, TNFRSF4, TNFRSF8, TNFRSF9, TNFRSF25, CD25, CD4, CD62L, CD27, CD70	[16–37]
	Inhibit proliferation	FOXP1, FOXO3, JNK2, VDR, IL-10R2, PD-1, TIM-3, CD38, CD160	[38–46]
	Inhibit function	PD-1, TIM-3, LAG-3, CTLA-4, TIGIT, T-bet, BLIMP1, NFAT, BATF, VHL, FOXO1, FOXP1, SLAMF6, TCF1, NR4A1, TOX, FGL1, B7H3, CD73, CD39, CD244, CD160	[47–65]
**CD4**^**+**^**T cell**	Control differentiation	IL2Rα, IL-4R, IL-6R, IL-10R, IL-12R, IL-13R, IL-21R, IL-23R, IL-25R, STAT1, STAT4, STAT6, GATA3, PPARG, IKZF2, CXCR5, FOXO1, FOXP3, CD8α, CD103, USP22, BRD9, RNF20, IRF4, CIC, PRDM1, TBX21, SATB1, HIVEP2, HDAC6, BCL6	[66–86]
**NK cell**	Promote proliferation	IL-2, IL-4, IL-12, IL-10, IL-15, IL-18, IL-21, IL-15Rα, CD16A, KLF2, TNFRSF4	[87–97]
	Inhibit proliferation	CD2	[98]
	Enhance toxicity effect	NKG2D, TNFRSF9, GRAIL, CD16, CD244, NTB-A, CS1, SCF	[99–104]
	Inhibit function	LAG-3, PD-1, TIM-3, TIGIT, KLRG1, KIR, NKG2A, CD96	[105–109]
**TAF**	Activate	IL-1R1, FGFR, GPER, TGFR, TNFR, GFs, VDR, NF-κB, JAK, STAT3, NLRP3, YAP, TAZ	[110–118]
	Regulate immune microenvironment	TGFβ, CCL2, CCL5, CCL7, CCL16, CXCL1, CXCL2, CXCL8, CXCL12, G-CSF, LIF, IL-6, IL-11, IL-33, NOX4, M-CSF, PGE2	[110, 119–127]
	Promote tumor growth, migration, invasion and epithelial-mesenchymal transformation	HGF, FGF1, PDGF, POSTN, OPN, CTGF, FOXF1, IL-17A, Cav1, FAP, α-SMA, FN1, VEGF, MMPs, MFAP5, ET-1	[122, 128–142]
	Inhibit tumor growth	ISLR, WFDC1	[143, 144]
**TAM**	Polarize to M1	TLR, DNMT3b, JMJD1A, HDAC3, HDAC9, STAT1, NF-κB, IRF5, Notch signaling, ERK5, MGLL, IRF1, IRF5, IRF8	[145–151]
	Polarize to M2	CSF-1R, CCR2, IL-1R, IL-4R, IL-10, IL-12R, IL-13R, IL-18R, GPR132, PRMT1, SMYD3, JMJD3, SIRT, BET, STAT3, STAT6, MYC, IRF3, IRF4, KLF4, PPARγ, COX-2, PI3Kγ	[146, 148, 152–160]

### Immune cells

Immune cells are the primary effectors involved in eliminating tumor cells. The primary target cells for gene editing in the TME are T cells, natural killer (NK) cells, and macrophages.

#### CD8^+^ T cells

T cells coordinate multiple aspects of adaptive immunity throughout life, including responses to pathogens, allergens, and tumors. They are classified as CD8^+^ and CD4^+^ T cells based on their expression of CD8 or CD4 molecules, respectively. CD8^+^ T cells are the main subset that directly kill tumor cells in the TME. Their infiltration is correlated with prognosis in several solid tumors [161, 162]. However, long-term antigenic stimulation in the TME causes CD8^+^ T cells to be unable to proliferate effectively and function persistently, preventing them from killing tumor cells [163]. Therefore, gene editing strategies aim to restore or enhance these two aspects of CD8^+^ T cells in the TME.

CD8^+^ T-cell proliferation is mainly stimulated by cytokines. Therefore, cytokine receptors on the cell surface are primary targets for gene editing. Cytokine receptors can be divided into two categories: those expressed in a variety of cells and those expressed in specific cells. Interleukin (IL)-2 was the first essential cytokine identified to maintain the survival and growth of T cells in vitro [164]. It has been used as a clinical cancer therapy. However, the pleiotropic properties of IL-2 cause severe toxicity due to the low specificity of IL-2 receptor (IL-2R) [164–168]. IL-2Rα on CD8^+^ T cells can be edited to bind to mutant IL-2 precisely [169]. Thus, intraperitoneal injection of mutant IL-2 was shown to specifically promote the proliferation of gene-edited CD8^+^ T cells in mice. This approach reprograms CD8^+^ T cells to be specifically stimulated to proliferate. Increasing IL-2 accumulation in the TME via fusion of IL-2 with tumor-targeting molecules is another way to explicitly promote CD8^+^ T-cell proliferation and reduce toxic side effects [170]. The second category of receptors can be artificially expressed in effector CD8^+^ T cells to promote cell proliferation specifically. For example, effector CD8^+^ T cells can be artificially engineered to express IL-7Rα. These reprogrammed cells can proliferate effectively in response to IL-7 stimulation both in vivo and in vitro, even in the presence of regulatory T cells (Tregs) [171]. Compared with IL-2-based approaches, these strategies can precisely promote the proliferation of effector CD8^+^ T cells and reduce cytokine-induced side effects by taking advantage of receptor specificity. In addition to IL-7, IL-15 and IL-21 specifically promote memory cell proliferation and are also candidates for gene editing [172, 173].

The ability of CD8^+^ T cells to persistently function is mainly limited by T-cell exhaustion due to prolonged antigenic stimulation. Exhausted CD8^+^ T cells are characterized by the loss of effector functions resulting from the upregulation of inhibitory receptors, such as programmed cell death 1 (PD-1), hepatitis A virus cellular receptor 2 (TIM-3), lymphocyte activating 3 (LAG-3), cytotoxic T-lymphocyte associated protein 4 (CTLA-4), T-cell immunoreceptor with Ig and ITIM domains (TIGIT) and other immune checkpoints [171, 174]. Gene editing can reprogram CD8^+^ T cells to maintain function in two ways: inhibiting exhaustion development and restoring or enhancing exhausted cell function. The development of exhaustion in CD8^+^ T cells is regulated by several transcription factors, including T-bet, Eomes, Blimp1, NFAT, BATF, VHL, FOXO1, FOXP1, TCF1, nuclear receptor subfamily 4 group A (NR4A), IRF4, and thymocyte selection-associated high mobility group box (TOX) [47, 48, 175–177]. Altering the expression of these factors can reduce CD8^+^ T-cell exhaustion [178, 179]. For example, TOX is a recently identified vital transcription factor that promotes CD8^+^ T-cell exhaustion. It functions in cooperation with NR4A [176]. CD8^+^ T cells from mice with TOX or NR4A knocked out were transplanted into tumor-bearing mice and showed reduced exhaustion [49, 180]. Controlling the expression of transcription factors via gene editing allows CD8^+^ T cells to remain functional and effectively destroy tumor cells. On the other hand, gene editing can be used to restore exhausted CD8^+^ T-cell functions by eliminating inhibitory receptors or reversing inhibitory receptor signaling. The most widely studied inhibitory receptor is PD-1. Knocking out PD-1 in CD8^+^ T cells with the CRISPR/Cas9 system was demonstrated to have antitumor effects in several preclinical and clinical studies, including studies on cancers including melanoma, glioblastoma, ovarian cancer, prostate cancer, B-cell lymphoma, gastric cancer, and breast cancer [181–189]. In addition, gene editing can be used to reverse inhibitory signaling. CD28 is a founding member of the costimulatory molecule subfamily and plays a role in amplifying TCR signaling [190]. Fusing PD-1 expressed by CD8^+^ T cells to CD28 via CRISPR/Cas9 gene editing reverses the original inhibitory signaling to achieve stimulatory cell signaling. This reprogramming strategy ultimately restores the effector function of exhausted CD8^+^ T cells [187–189].

#### CD4^+^ T cells

Although CD8^+^ T cells are currently the most studied T cells, a large number of studies have shown that CD4^+^ T cells also have essential functions in the TME [191]. CD4^+^ T cells can differentiate into different subtypes. Their roles in the TME are different or even opposite in terms of immunity. CD4^+^ T helper cells and Tregs are two major subtypes. CD4^+^ T helper cells help regulate the gene expression profiles of CD8^+^ T cells to enhance tumor-eliminating effects [192]. In contrast, Tregs act as immune suppressors [193].

Two strategies can be employed to reprogram CD4^+^ T cells using gene editing: controlling their differentiation into helper cells and inhibiting Treg function. Currently, the genes known to influence the differentiation fate of CD4^+^ T cells mainly include IL2Rα, PPARG, and IKAROS family zinc finger 2 (IKZF2) [66, 194, 195]. Based on function, different strategies should be used. For example, knocking out the IL2Rα enhancer with CRISPR/Cas9 reprograms CD4^+^ T cells to differentiate from naive cells into Th17 cells [195]. In contrast, knocking out IKZF2 via CRISPR/Cas9 reprograms human fetal naive CD4^+^ T cells to differentiate into Tregs [194]. Gene editing could be designed to induce CD4^+^ T cells to differentiate into cells that promote immune responses.

Furthermore, gene editing has been utilized to reverse the immunosuppressive effects of Tregs [196]. This type of cell is characterized by high expression of forkhead box P3 (FOXP3), which plays a vital role in immunosuppressive functions [197]. Loss of FOXP3 function is associated with autoimmunity in both humans and mice [198]. Knocking out FOXP3 in Tregs via gene editing is beneficial for promoting an immune response in the TME. Therefore, identifying genes upstream of FOXP3 via gene editing technology can help reverse the immunosuppressive phenotype. Several studies have used CRISPR screening to identify upstream regulators of FOXP3, including ubiquitin specific peptidase 22 (Usp22), bromodomain containing 9 (Brd9), and Rnf20 [67, 68]. Knocking out Usp22 and Brd9 was shown to reduce FOXP3 expression and impair the immunosuppressive function of Tregs in mice. The CRISPR/Cas9 system can be employed in this strategy to reprogram the immunosuppressive effect of Tregs and ultimately inhibit tumor growth. Other FOXP3 regulators identified using the CRISPR library include FOXO1, IRF4, GATA3, CIC, PRDM1, TBX21, SATB1, and HIVEP2 [199]. They provide several targets for Treg reprogramming.

#### NK cells

NK cells can kill tumor cells directly, showing better safety than CD8^+^ T cells with minor cytokine release syndrome (CRS) and neurotoxicity [200–202]. The limitations related to the clinical use of NK cells are weak proliferation and cytotoxicity. Given these two points, gene editing can reprogram NK cells to promote their effective proliferation and persistent functionality.

The proliferation of NK cells is mainly regulated by the cytokines IL-2, IL-12, IL-15, IL-21, and IL-18 [203]. Among these cytokines, IL-15 is recognized to have essential roles in cell survival and proliferation. Gene editing can be used to edit IL-15 in NK cells to promote NK-cell proliferation in vivo. NK cells overexpressing IL-15 exhibit both a stronger proliferative ability and potential side effects [204]. Therefore, membrane-bound IL-15 (mbIL-15) was developed. This protein is a fusion protein of IL-15 and the NK-cell CD8α transmembrane structural domains located on the cell membrane [205]. This engineering allowed mbIL-15 to stimulate adjacent NK cells without inducing the side effects caused by free cytokines. This strategy has the potential to maintain NK-cell proliferation.

The toxic effect of NK cells can be enhanced by promoting their toxic effects or restoring the toxic effects of inhibitory cells. NK92 cells are an NK cell line with an indefinite proliferative ability that are widely used in clinical trials [206, 207]. However, they exhibit lower toxicity to tumor cells than primary NK cells, which has limited their development. Gene editing can be used to overcome this challenge. Antibody-dependent cell-mediated cytotoxicity (ADCC) is one of the most critical mechanisms by which NK cells kill tumor cells. CD16 expressed on NK cells recognizes the Fc portion of IgG bound to the tumor cell surface and eliminates tumor cells through ADCC [207]. Therefore, CRISPR/Cas9 can be used to reprogram NK cells to improve the ADCC effect by overexpressing CD16 [208]. On the other hand, the function of NK cells is limited by the activation of inhibitory receptors. Knocking out these receptors via gene editing can restore cell function. The primary identified inhibitory receptors of NK cells are LAG-3, PD-1, TIM3, and TIGIT [209]. TIGIT is a newly identified shared inhibitory receptor in exhausted CD8^+^ T and NK cells. Moreover, TIGIT but not CTLA-4 or PD-1 is associated with NK-cell exhaustion in tumor-bearing mice and colon cancer patients [105]. CRISPR/Cas9 has been used to specifically knock out TIGIT in mouse NK cells. The results showed that these cells exhibited restored cytotoxicity and killing ability specific for tumor cells [105]. Similar to TIGIT, other inhibitory receptors on NK cells can be knocked out to restore the tumor-killing function of these cells.

#### Tumor-associated macrophages (TAMs)

TAMs are macrophages in the TME. They are a double-edged sword for tumor cells. Cytokines can polarize TAMs into M1 or M2 macrophages that exhibit anticancer or procancer functions, respectively. There are two strategies to reprogram TAMs via gene editing: polarizing them into M1 macrophages and promoting M2 macrophage death. TAM polarization into M1 macrophages is mainly regulated by the cytokines IL-9, IL-27, and IL-12 [210–212]. Upregulation of these cytokines by gene editing promotes M1 macrophage polarization. It was reported that high expression of IL-12 in TAMs results in a more than four-fold increase in the M1/M2 macrophage ratio [212]. In addition, regulating upstream signaling pathways of IL-2, including the signal transducer and activator of transcription 3 (STAT3)/NF-Kappab/C-REL and inhibitor of nuclear factor-kappa B kinase subunit beta (IKKβ)/NF-Kappab signaling pathways, can also affect the TAM polarization direction. Knocking out STAT3 or IKKβ in TAMs via gene editing was shown to induce M1 macrophage polarization and effectively inhibit tumor growth in mice [213, 214]. TAM polarization into M2 macrophages is mainly related to the activation of colony-stimulating receptor (CSF1R) and C-C motif chemokine receptor 2 (CCR2) on the cell surface. Knocking out CSF1R repolarizes M2 macrophages into M1 macrophages and enhances phagocytic activity [215]. Current phase I and II clinical trials of drug therapies targeting CSF1R in giant cell tumors have yielded promising results [216]. However, serious side effects were observed in patients. Blocking the CCL2/CCR2 signaling pathway via gene editing results in TAM polarization into M1 macrophages and promotes antitumor immune responses in various mouse models, including lung, esophageal, and liver cancer models [217–219].

### Nonimmune cells

Nonimmune cells in the TME are culprits in tumorigenesis, providing nutrition and energy for tumor cells. There are many nonimmune cells in the TME, including tumor-associated fibroblasts (TAFs), endothelial cells, mesenchymal stem cells, and adipocytes. Currently, gene editing is mainly used to reprogram TAFs, which are the main focus of our discussion.

#### TAFs

TAFs are significant components of the TME cell population in solid tumors [220, 221]. They are heterogeneous and act as either the foundation or walls of tumors. Depending on their roles in tumors, they can be classified into cancer-promoting TAFs and cancer-suppressing TAFs [222, 223]. The former promotes tumor progression dependent on IL-1R activation and the subsequent release of inflammatory factors, including TSLP, IL-6, and CXCL12 [223]. The latter may inhibit tumor progression by remodeling the collagen structure [143]. Therefore, gene editing can reprogram TAFs to inhibit tumor progression by inhibiting the function of cancer-promoting TAFs or enhancing the function of cancer-suppressing TAFs.

For cancer-promoting TAFs, gene editing aims to inhibit their activation. IL1/IL-1R is essential for activating cancer-promoting TAFs and promotes the release of proinflammatory factors via the activation of the JAK/STAT3, PI3KCA/AKT, and NF-κB signaling pathways [224–226]. Therefore, gene editing can reprogram TAFs by blocking IL-1R activation and reducing the secretion of inflammatory factors. In a study, fibroblasts with or without IL-1R1 knocked out and breast cancer cells were coimplanted into the lateral abdomen of BALB/c mice. Compared with the WT fibroblast group, the Il1r1^−/−^ fibroblast group showed inhibition of tumor cell growth following coimplantation [225]. In addition, TAFs have been shown to have a reduced proinflammatory phenotype when the downstream IL1/IL-1R pathway (JAK/STAT3 and PI3KCA/AKT) is inhibited.

For cancer-suppressing TAFs, gene editing can alter the expression of genes to increase their tumor-suppressive ability. Cancer-suppressing TAFs have been poorly studied. To date, it has been found that TAFs expressing immunoglobulin superfamily containing leucine rich repeat (ISLR) or Caveolin-1 (CAV-1) can inhibit tumor progression [143, 227]. ISLR was the first identified marker of cancer-suppressing TAFs in human and mouse pancreatic ductal carcinoma (PDAC). High expression of ISLR in TAFs correlates with a good patient prognosis [143]. Ablation of TAFs expressing ISLR in mouse models leads to malignant progression, while exogenous expression of ISLR inhibits tumor progression. ISLR is a potential therapeutic target for reprogramming TAFs for cancer therapy. The mechanism of tumor growth inhibition mediated by CAV-1-expressing fibroblasts is unclear. The functional mechanism of tumor-suppressive TAFs needs to be further studied.

#### Other cells

In addition, gene editing can also reprogram other cells, such as B cells and dendritic cells (DCs). B cells produce membrane-bound or secretory immunoglobulins in lymphoid tissues or plasma. At present, researchers mainly use gene editing to induce B cells to express antibodies [228, 229]. Since tumor cells often evade host immunity by expressing inhibitory receptors, gene editing can reprogram B cells to express monoclonal antibodies. This is a potential approach for cancer treatment, as these antibodies can competitively bind to inhibitory receptors. DCs are special antigen-presenting cells, and their costimulatory signaling molecule CD40 is critical in regulating T-cell activation and promoting graft rejection. Knocking out CD40 in DCs via CRISPR/Cas9 prevents transplant rejection, which is one of the barriers to adoptive therapy for cancers [230]. Many other cell types have not been thoroughly studied. They are also potential target cells for gene editing.

## Reprogramming cell–cell communication

**Fig. 4:**
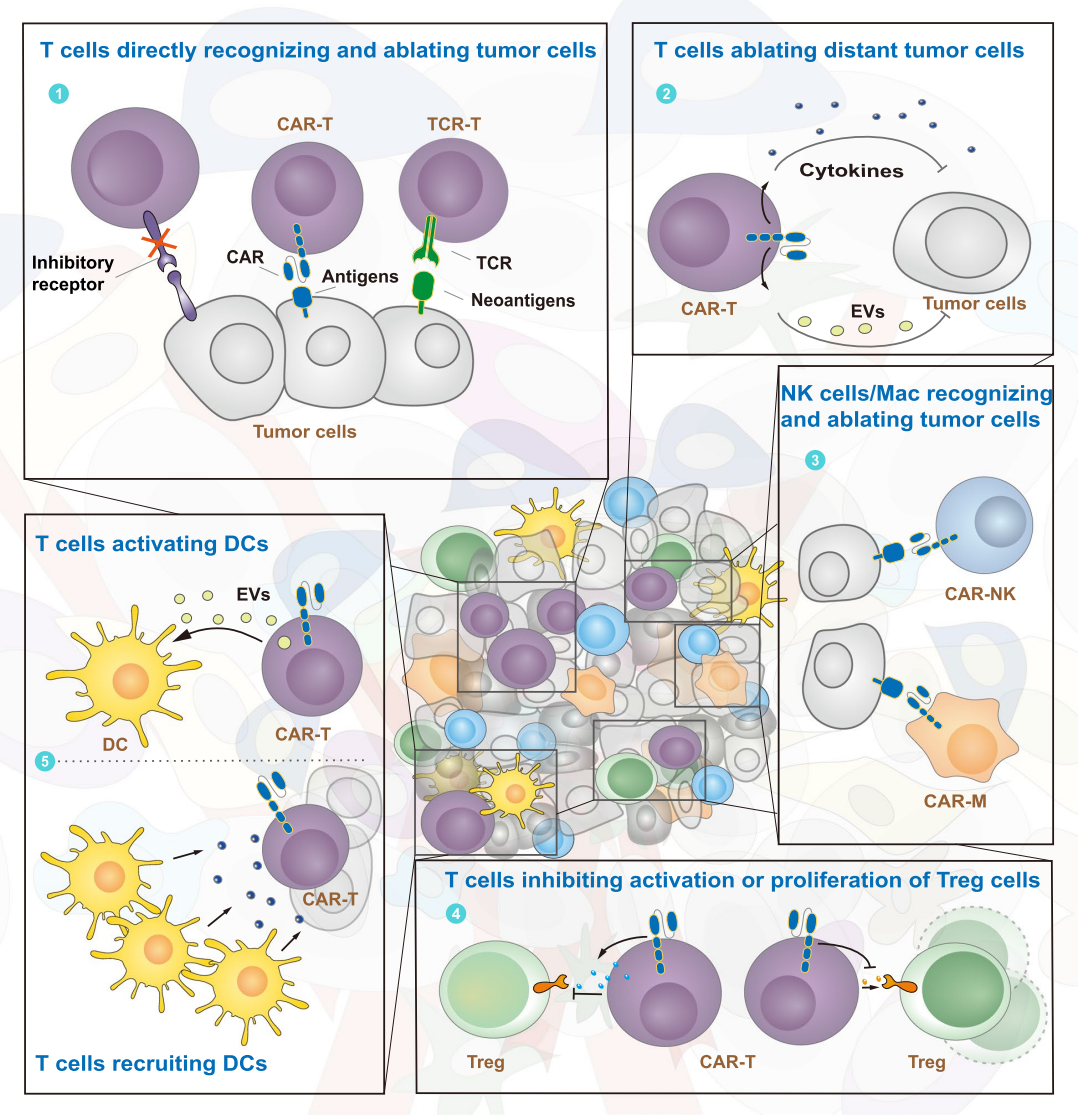
Reprogramming TME intercellular communication via gene editing. Gene editing is used to reprogram TME intercellular communication, including communication between tumor cells and immune cells or between different immune cells. The former includes T cell–tumor cell, NK cell–tumor cell, and macrophage–tumor cell communication, while the latter includes T cell–DC and T cell–Treg communication. The application of gene editing in immune cell–tumor cell communication facilitates enhancement or restoration of the ability of immune cells to recognize tumor cells. In immune cell–immune cell communication, gene editing is mainly used to promote antigen presentation by DCs and inhibit the immunosuppressive activity of Tregs. As shown in the panels, ①-③ show communication between tumor and immune cells, and ④⑤ show communication between different immune cells

Complex intercellular communication among TME cells provides inhibitory or stimulatory signals that influence tumor cell fate [231]. Therefore, the effectiveness of tumor cell killing by immune cells is determined by the intrinsic properties of both cell types and is intimately associated with intercellular communication. Gene editing provides a flexible and safe tool to reprogram TME intercellular communication for cancer therapy (Fig. 4). It is becoming a focus in tumor immunotherapy.

### Immune cell–tumor cell communication

#### T cells and tumor cells

Intercellular communication between T cells and tumor cells is the most studied type of intercellular communication. It includes adjacent cell–cell communication through recognition between receptors and ligands on the cell surface and distant communication through secreted mediators (cytokines, chemokines, adhesion molecules, and exosomes). At present, gene editing is more widely used to modulate the former. The Food and Drug Administration (FDA) has approved genetically modified T cells as drugs for the treatment of tumors, including tisagenlecleucel (Tisa-cel), axicabtagene ciloleucel (Axi-cel), lisocabtagene maraleucel (Liso-cel), and brexucabtagene autoleucel (Brexu-cel) [232–234]. This section will introduce how gene editing is used to reprogram cell–cell communication to eliminate tumors.

##### Adjacent communication

Adjacent cell–cell communication is dependent on physically adjacent structures or ligand–receptor interactions. The latter can be reprogrammed by gene editing. Currently, the most effective and promising application in this area is adoptive T-cell therapy (ACT) [232, 235]. ACT refers to isolating T cells from a patient, equipping the T cells with modified antigen recognition receptors via gene editing, and reinfusing them into the patient’s body after expansion. According to the autologous or allogenic modified antigen recognition receptor introduced, T cells utilized for ACT can be divided into chimeric antigen receptor T (CAR-T) cells and recombinant T-cell receptor T (TCR-T) cells [236].

CAR-T cells are designed to recognize cancer cells that escape surveillance by unmodified T cells. They bind to specific antigens on the tumor cell surface; secrete the cytokines IL-12, IL-15, and IL-18; and then recognize and eliminate tumor cells [237]. The CAR consists of extracellular recognition, transmembrane, and intracellular signaling domains. Modifying the CAR extracellular domain via gene editing enables T cells to recognize antigens on tumor cells. Cancer cells in tissues are highly heterogeneous. The antigens on the cell surface may differ, or several antigens may be expressed simultaneously. The elasticity of gene editing makes the antigen recognition mode of CAR-T cells flexible, thus promoting clinical application. The main target antigens and applied tumor types for CAR-T cells are shown in Table 3. Among these antigens, CD19 is the most important and studied. It is evenly distributed in malignant B cells and considered a prime target for T-cell recognition [238]. Gene-edited CAR-T cells targeting CD19 can identify and eliminate tumor cells [239]. Compared with single antigen-recognition, multiple-antigen recognition increases the affinity of CAR-T cells for tumor cells. These CAR-T cells can recognize tumor cells expressing various antigens individually or simultaneously. Gene editing has supported the generation of second-, third-, and fourth-generation CAR-T cells. In addition to CD19, CD22 is widely distributed on the B-cell surface in most cases of B-cell acute lymphoblastic leukemia (B-ALL) [240]. These antigens can exist on the same or different tumor cell surfaces. A tandem CAR was developed and introduced to T cells to target these two antigens. CD19-CD22 CAR-T cells exhibited better tumor elimination than CD19 CAR-T cells in patient-derived xenograft (PDX) models [241]. Compared with previous approaches, CAR recognition of three antigens has further improved the recognition ability and affinity. A typical example is as follows. Human epidermal growth factor receptor 2 (HER2), interleukin-13 receptor subunit alpha-2 (IL13Rα2), and ephrin-A2 (EphA2) are specifically expressed on the surface of glioblastoma, recurrent medulloblastoma, and ependymoma cells [242]. CAR-T cells targeting these three antigens can recognize and eliminate tumor cells in PDX mouse models [242, 243].

**Table 3:** Targeted antigens of CAR-T cells, CAR-NK cells and CAR-Ms in cancers

Cell Type	Target antigen	Application	Reference
CD8+ T cell	CD19	Acute Lymphoblastic Leukaemia, Multiple Myeloma, B-Cell Lymphoma	[244–247]
	CD20	Melanoma, Diffuse Large B-Cell Lymphoma, Non-Hodgkin Lymphoma, Burkitt Lymphoma	[247–251]
	CD22	B Acute Lymphoblastic Leukemia, Burkitt Lymphoma	[251, 252]
	CD30	Hodgkin’s Lymphoma	[253]
	CD33	Acute Myeloid Leukemia	[254]
	CD56	Rhabdomyosarcoma	[255]
	CD70	Renal Carcinoma, B-Cell Lymphoma	[256–258]
	CD133	Cholangiocarcinoma, Hepatocellular Carcinoma, Pancreatic Carcinomas, Colorectal Carcinomas	[259, 260]
	CD138	Multiple Myeloma	[261]
	CD171	Neuroblastoma	[262]
	HER2	Biliary Tract Cancer, Pancreatic Cancers	[263]
	EGFR	Non-Small Cell Lung Cancer, Cholangiocarcinoma, Biliary Tract Cancers, Pancreatic Carcinoma	[259, 264–266]
	MSLN	Gastric Cancer, Pancreatic Cancer, Pleural Mesothelioma, Ovarian Carcinoma, Biliary Tract Cancer, Tubal Cancer, Esophageal Cancer, Cervical Cancer, Triple-Negative Breast Cancer	[189, 267–270]
	LMP1	Lymphoma, Nasopharyngeal Carcinoma	[271, 272]
	FR-α	Ovarian Carcinoma, Colorectal Carcinomas, Pancreatic Cancer, Lung Cancer	[273]
	EGFRIII	Glioblastoma	[274, 275]
	GPC3	Hepatocellular Carcinoma, Pancreatic Carcinoma, Ovarian Carcinoma	[276, 277]
	PSCA	Chronic Myelogenous Leukemia, Gastric Cancer	[278, 279]
	MUC1	Lung Cancer, Seminal Vesicle Cancer,	[280, 281]
	MAGE-A1/3/4	Lung Adenocarcinoma	[282]
	EPCAM	Chronic Myelogenous Leukemia, Breast Cancer, Lung Cancer, Acute Myeloid Leukemia, Colorectal Cancer	[283–286]
	PSMA	Prostate Cancer	[287]
	AXL	Breast Cancer	[288]
	MUC16	Ovarian Cancer	[289]
	DR5	B-Cell Malignancies	[290]
	c-MET	Hepatocellular Carcinoma, Gastric Cancer, Renal Cell Carcinoma	[291–293]
	BCMA	Multiple Myeloma	[294–296]
	GPC3	Hepatocellular Carcinoma	[297]
	CS1/SLAMF7	Multiple Myeloma,	[298]
	NKG2D	Hepatocellular Carcinoma, Glioblastoma	[299, 300]
	CLL-1	Acute Myeloid Leukemia	[301, 302]
	CEA	Colorectal Cancers, Pancreatic Malignancy, Hepatocellular Carcinoma	[270, 303, 304]
NK cell	CD5	T Cell Malignancies	[305]
	CD7	Lymphoma, Leukemia	[306]
	CD19	Acute Lymphoblastic Leukaemia, Chronic Lymphocytic Leukemia, B Lymphoblastoid, Monocytic Leukemia, Ovarian Cancer, Chronic Myelocytic Leukemia, Breast Cancer, Lung Cancer, Gastric Cancer, Epidermoid Carcinoma, Bladder Cancer	[201, 307, 308] [309–311]
	CD20	B-Cell Malignancies, Burkitt Lymphoma	[312, 313]
	CD33	Acute Myeloid Leukemia.	[314]
	CD38	Acute Myeloid Leukemia.	[315]
	CD123	Acute Myeloid Leukemia, Blastic Plasmacytoid Dendritic Cell Neoplasm	[316–318]
	CD138	Multiple Myeloma	[319]
	CS1	Ovarian Cancer, Multiple Myeloma	[320, 321]
	EBNA3C	Leukemia	[322]
	EGFRvIII	Glioblastoma	[323]
	EPCAM	Breast Carcinoma	[324]
	GD2	Neuroblastoma, Ewing Sarcomas,	[325, 326]
	GPA7	Melanoma	[327]
	GPC3	Ovarian Cancer	[328]
	HER-2	Glioblastoma, Breast Cancer, Renal Cell Carcinoma	[329–331]
	HLA-A2	Melanoma	[327]
	HLA-DR	Glioblastoma	[332]
	HLA-G	Leukemia	[333]
	MSLN	Ovarian Cancer	[334]
	PSCA	Ladder Carcinoma	[335]
Macrophage	HER2	Chronic Myelocytic Leukemia	[309]
	MSLN	Chronic Myelocytic Leukemia	[309]

TCR-T cells are cells with a modified endogenous TCR antigen recognition domain designed to enhance the recognition of tumor cells by T cells [15]. They have been mainly used to recognize the mutation-derived neoantigens of cancer cells. TCR-T cells recognize specific antigens presented in linear 8–11 amino acid peptides presented by MHC class I. Thus, TCR-T cells can recognize peptides derived from an entire cell, including the cell surface, cytoplasm, and nucleus. Gene editing can be used to modify the endogenous TCR antigen recognition domain to recognize a mutant peptide derived from a neoantigen. Currently, nearly 200 clinical trials are evaluating the safety or effectiveness of TCR-T cell therapy. The most commonly targeted and promising cancer cell antigen is NY-ESO-1. NY-ESO-1^c259^-specific TCR-T cells were produced with the goals of recognizing and eliminating antigen-positive tumor cells [15, 336]. TCR-T cell treatment has shown a relatively good clinical effect. Twelve recurrent or metastatic synovial sarcoma patients received NY-ESO-1^c259^ TCR-T cell treatment, tumors shrank significantly in half of the patients, and no fatal severe adverse events occurred [337]. Similarly, TCR-T cells recognizing the MyD88^L265P^ mutation can target tumor cells carrying this mutation in B-cell malignancies [338]. In addition to cytoplasmic antigens, membrane antigens can be recognized by TCR-T cells. The most studied antigen is mesothelin (MSLN). Compared with TCR-T cells targeting other epitopes, TCR-T cells specifically targeting Msln^406–414^ epitopes show relatively high affinity for tumor cells in pancreatic ductal adenocarcinomas (PDAs).

During ACT treatment, a considerable amount of tumor tolerance is observed. Immune escape mediated by immune checkpoints is recognized as one of the main reasons. To promote immune escape, immune checkpoint molecules expressed on the tumor cell membrane bind to paired receptors on the surface of immune cells. Knocking out immune checkpoint molecules in CAR-T and TCR-T cells with CRISPR/Cas9 technology allows these cells to recognize escaped tumor cells and restores intrinsic recognition. Compared with immune checkpoint inhibitors, gene editing targets specific immune cells and does not require systemic immune blockade or induce immune-related side effects [339]. In addition, according to the individual differences among patients, gene editing can knock out one or multiple immune checkpoint genes to achieve personalized immunotherapy. PD-1 and CTLA4 are the most studied checkpoint molecules. In a refractory pan-cancer dataset, knocking out PD-1 improved the recognition of tumor cells by NY-ESO-1^c259^ TCR-T cells [15]. Similarly, in CD19 CAR-T cells, PD-1 knockout significantly improved the recognition of tumor cells in refractory non-small cell lung cancer, lymphoma, and chronic myelogenous leukemia [14]. In acute lymphoblastic leukemia (ALL) and bladder cancer, knocking out CTLA-4 augmented recognition by T cells. In addition, gene editing can simultaneously inhibit the expression of multiple immune checkpoint molecules via knock out of mutual regulators. For example, nuclear factor of activated T cells (NFAT) is a key transcription factor regulating T-cell activation [340]. It increases the expression of multiple inhibitory receptors, including PD1, LAG3, TIM-3, and GITR, on the cell surface. Knocking out NFAT using gene editing was shown to significantly inhibit the expression of these inhibitory receptors in vivo [50].

##### Distant communication

Distant T cells and tumor cells can communicate through mediators, including cytokines, chemokines, adhesion molecules, and extracellular vesicles (EVs). These factors can also be reprogrammed. However, gene editing strategies targeting these factors are still in the preclinical phase. Among them, the most studied target is EVs. EVs are nanoscale vesicles secreted by almost all cells and contain bioactive molecules. They transmit information from donor to recipient cells and participate in physiological and pathological processes. In recent years, they have been found to regulate the TME and affect immune cell functions [341, 342].

Gene editing can be used to edit cells to produce attractive substrates that can be delivered by EVs and enhance EV targeting. Gene editing can be used to add genes encoding CAR-targeting antigens to traditional CAR molecules, allowing CAR-T cells to express such antigens. These antigens are then packaged into EVs and delivered to tumor cells. Specifically, the EVs localize at the tumor cell membrane and deliver antigens to the tumor cells. Then, the target tumor cells develop increased antigen expression on the cell surface. In this way, CAR-T cells can recognize tumor cells without expression or with low expression of antigens [343]. Gene editing can directly modify EVs derived from T cells so that cargo can be more accurately packaged into the EVs. The tetraspanin CD9 is a marker molecule located on the EV membrane [344]. In T cells, genetic fusion of CD9 with other proteins can better enrich the target molecules in EVs. Then, these molecules can play a role in suppressing tumors after the EVs reach the target cells. For example, fusing CD9 with HuR by gene editing can enrich HuR-binding RNAs in EVs. These RNAs reach target cells in the EVs and kill tumor cells [345]. Similarly, fusing CD9 with PhoCl can achieve light-controlled release of cargo proteins after arrival. Fusion of CD9 with CD70 can successfully localize CD70 on the surface of target cells and thus provide costimulation to T cells [346]. In addition to EVs, other factors secreted by CAR-T cells, such as IL-12, IL-15, and IL-18, play roles in killing tumor cells [347].

#### NK cells and tumor cells

In the clinic, CAR-T cell treatment is limited by graft-versus-host disease (GVHD) and the long production cycle. Thus, CARs can be introduced into other immune cells as well. Among these cells, NK cells are most commonly used. Compared with T cells, NK cells have a more comprehensive tumor recognition range and more robust antitumor function. The lack of TCR expression by NK cells prevents them from causing GVHD. The assembly of antigen recognition receptors on the surface of NK cells can enable CAR-engineered NK (CAR-NK) cells to recognize tumors more accurately and exert a powerful tumor-killing effect. The development of CAR-NK cells is attracting significant attention.

Gene editing can reprogram NK cells to increase specific recognition and remove inhibitory immune checkpoint molecules on the surface. Most of the CARs that have been introduced into NK cells were designed for CAR-T cells. Working from traditional CAR structures, using gene editing to replace 4-1BB/CD28 with 2B4 (an NK cell-specific costimulatory domain), CARs specifically designed for NK cells can be obtained. The main target antigens and applied tumor types for CAR-NK cells are shown in Table 3. For these antigens, the most studied CARs are those recognizing CD19 and CD5. NK cells equipped with CD19 showed powerful recognition and a strong killing effect against CD19^+^ relapsed or refractory tumors. Eight patients showed remission among the 11 lymphoid patients who received treatment [201]. Moreover, due to the lack of TCR expression and IL-6 release, patients who received CAR-NK allografts did not develop CAR-T cell-related serious toxic effects, including neurotoxicity, cytokine release syndrome, and GVHD [201]. CD5 is highly expressed in malignant T cells and considered one of the characteristic antigens of malignant T cells [348]. In this case, due to the similarity between normal and malignant T cells, CD5 CAR-T cells may produce fratricide and cause normal T-cell hypoplasia. CD5 CAR-NK cells can be used to accurately recognize CD5^+^ tumor cells and prolong T-cell acute lymphoblastic leukemia (T-ALL) xenograft mouse survival [305]. Other similar targets include CD20, CD123, GPC3, MSLN, CD38, CD147 and EGFR [312, 315, 349]. Gene editing can modify them to produce the corresponding CAR-NK cells to eliminate tumors. However, CAR-NK cells are still in the preclinical research stage. Inhibitory immune checkpoint molecules, such as PD-1 and TIGIT, are also expressed on the surface of NK cells and inhibit their recognition activity. In colon cancer, by knocking out these inhibitory immune checkpoint molecules, CRISPR/Cas9 technology restores the recognition ability of NK cells and promotes NK cell-dependent antitumor immunity [105].

#### Macrophages and tumor cells

In most cancers, macrophages are widely distributed in the TME. Compared with other immune cells, macrophages can penetrate tumor tissues more readily. The lack of TCR expression prevents macrophages from causing GVHD. In addition, macrophages perform phagocytosis and antigen presentation and exhibit cytotoxic activity [350]. While the recognition function of macrophages is nonspecific, equipping macrophages with CARs via gene editing can increase their recognition of tumor cells. The main target antigens and applied tumor types for CAR macrophages (CAR-Ms) are shown in Table 3. In addition, gene editing can be used to enhance phagocytosis by macrophages.

Primarily, gene editing can be used to increase the recognition of tumor cells. Similarly, the most important and studied CAR introduced into macrophages is the CD19 CAR. For instance, CD19 CAR-Ms were shown to decrease the tumor burden and prolong overall survival in solid tumor xenograft mouse models [309]. In addition, MSLN is another common molecule exploited in gene editing. MSLN is highly expressed in mesothelioma, pancreatic adenocarcinoma, ovarian cancer, and lung adenocarcinoma [349]. CAR-Ms targeting MSLN show increased phagocytic activity against ovarian/pancreatic cancer cells expressing MSLN [310].

CARs for phagocytosis (CAR-Ps) can be introduced into macrophages to enhance phagocytosis. For example, multiple EGF-like domains (Megf10) and an Fc receptor (FcRɣ) robustly trigger phagocytosis in macrophages. Inclusion of Megf10 and FcRɣ in CD19 CAR-Ms vastly enhances their phagocytic ability [311]. An additional tandem PI3K recruitment domain further promotes the phagocytosis of tumor cells.

### Immune cell–immune cell communication

Interactions among different immune cells in the TME exert immunostimulatory or immunosuppressive effects. Gene editing can be used to reprogram immune cell–immune cell communication to eliminate tumors. However, there are only a few investigations in this area.

#### T cells and Tregs

Immunosuppression mediated by Tregs is an important cause of CAR-T-cell failure in clinical practice. Gene editing can be used to suppress the communication between effector T cells and Tregs to inhibit the immunosuppressive effect of Tregs. Conventional CAR-T cells secrete IL-2 upon antigen encounter, which leads to the generation of Tregs. CD28 induces the production of IL-2, while mutant CD28 can inhibit the production of IL-2. Utilizing gene editing to substitute two amino acids in the PYAP Lck binding motif in the CD28 domain (ΔCD28) of CARs can inhibit the production of IL-2 and generation of Tregs [351]. In this way, decreasing Treg levels weakens their immunosuppressive effect. In addition, gene editing can be used to suppress Treg function. IL-12 is critical in suppressing the function of Tregs. When IL-12 is included in CAR molecules, CAR-T cells can secrete IL-12 to inhibit the suppressive function of Tregs. Significant Treg inhibition and tumor clearance have been observed in animal models of thymoma and glioblastoma [347, 352].

#### T cells and DCs

DCs are essential antigen-presenting cells. Most antigens are processed by DCs and then presented to T cells. Gene editing can be used to improve communication between T cells and DCs. For example, the highly structured noncoding RNA RN7SL1 can be introduced into CAR-T cells via gene editing. Then, RN7SL1 can be carried by exosomes to act on DCs and promote their activation and antigen-presenting functions [343]. In addition, factors released by gene-edited T cells can increase the infiltration of DCs. CCL19 and CD40L are important DC chemoattractants. Engineered T cells with inserted CCL19 or CD40L can release these molecules, increasing the infiltration of DCs into tumors [353, 354].

## Conclusion and perspective

In this review article, we summarize the application of gene editing for reprogramming TME cells and intercellular communication. In this way, gene editing promotes the killing effect of immune cells on tumor cells. Tumor tissues are highly heterogeneous, and the features of tumor cells and immune cells in the TME are very different even within a single tumor. In response to this heterogeneity, gene editing can accurately change the features of immune cells or tumor cells in a flexible and changeable way. The entire microenvironment is reprogrammed to become unsuitable for tumor survival. With the application of gene editing technology in epigenetics, epitranscriptomics, and proteomics, the methods for reprogramming the TME have expanded from traditional gene knock in and out strategies to making various modifications to genes, transcripts, and proteins. This means that cell reprogramming can be more diversified and accurate according to cell features. In addition, the number of cell types that can undergo gene editing has increased and now includes pluripotent stem cells and hematopoietic stem cells. To date, FDA-approved gene editing treatments are based on T cells. In short, with further improvements in the safety and effectiveness of gene editing, an increasing number of edited cell types will be used in the clinical treatment of tumors. Overall, gene editing can be used to reprogram the TME and promote precision treatment of tumors.

## Data Availability

Not applicable.

## Abbreviations

TME: Tumor microenvironment
MegaNs: Meganucleases
ZFNs: Zinc finger nucleases
TALENs: Transcription activator-like effector nucleases
CRISPR/Cas: Clustered regularly interspaced short palindromic repeats/ CRISPR-associated proteins
TALEs: Transcription activator-like effectors
iPSCs: Human induced pluripotent stem cells
CrRNA: CRISPR-derived RNA
NK cells: Natural killer cells
TCR: T cell receptor
IL-2: Interleukin 2
IL-2R: IL-2 receptor
IL-7: Interleukin 7
Tregs: Regulatory cells
IL-15: Interleukin 15
IL-21: Interleukin 15
PD-1: Programmed cell death 1
TIM-3: Hepatitis A virus cellular receptor 2
LAG-3: Lymphocyte activating 3
CTLA-4: Cytotoxic T-lymphocyte associated protein 4
TIGIT: T cell immunoreceptor with Ig and ITIM domains
NR4A: Nuclear receptor subfamily 4 group A
TOX: Thymocyte selection associated high mobility group box
IKZF2: IKAROS family zinc finger 2
FOXP3: Forkhead box P3
Usp22: Ubiquitin specific peptidase 22
Brd9: Bromodomain containing 9
CRS: Cytokine release syndrome
mbIL-15: Membrane-bound IL-15
ADCC: Antibody-dependent cell-mediated cytotoxicity
TAM: Tumor-associated macrophages
IL-12: Interleukin 12
STAT3: Transducer and activator of transcription 3
IKKβ: Inhibitor of nuclear factor kappa B kinase subunit beta
CSF1R: Colony-stimulating receptor
CCR2: C-C motif chemokine receptor 2
Axi-cel: Axicabtagene ciloleucel
TAF: Tumor-associated fibroblast
ISLR: Leucine rich repeat
CAV-1: Caveolin-1
PDAC: Pancreatic ductal carcinoma
DC: Dendritic cells
Tisa-cel: Tisagenlecleucel
Liso-cel: Lisocabtagene maraleucel
Brexu-cel: Brexucabtagene autoleucel
ACT: Adoptive T-cell therapy
CAR-T: Chimeric antigen receptor T cells
TCR-T: T-cell receptor T cells
B-ALL: B cell acute lymphoblastic leukemia
HER2: Human epidermal growth factor receptor 2
IL13Rα2: Interleukin-13 receptor subunit alpha-2
EphA2: Ephrin-A2
PDXs: Patient derived xenografts
MSLN: Mesothelin
PDAs: Pancreatic ductal adenocarcinomas
NFAT: Nuclear factor of activated T cells
EVs: Extracellular vesicles
GVHD: Graft-versus-host disease
CAR-iMac: Pluripotent stem cell-derived CAR-macrophage cells
CAR-Ps: CARs for phagocytosis
Megf10: Multiple EGF like domains
FcR: Fc receptor

## References

[1] Quail DF, Joyce JA (2013). Microenvironmental regulation of tumor progression and metastasis. Nat Med.

[2] Grunwald BT, Devisme A, Andrieux G (2021). Spatially confined sub-tumor microenvironments in pancreatic cancer. Cell.

[3] Luca BA, Steen CB, Matusiak M (2021). Atlas of clinically distinct cell states and ecosystems across human solid tumors. Cell.

[4] Chevalier BS, Stoddard BL (2001). Homing endonucleases: structural and functional insight into the catalysts of intron/intein mobility. Nucleic Acids Res.

[5] Kim H, Kim JS (2014). A guide to genome engineering with programmable nucleases. Nat Rev Genet.

[6] Beane JD, Lee G, Zheng Z (2015). Clinical scale zinc finger nuclease-mediated gene editing of PD-1 in tumor infiltrating lymphocytes for the treatment of metastatic melanoma. Mol Ther.

[7] Bukhari H, Muller T (2019). Endogenous fluorescence tagging by CRISPR. Trends Cell Biol.

[8] Joung JK, Sander JD (2013). TALENs: a widely applicable technology for targeted genome editing. Nat Rev Mol Cell Biol.

[9] Poirot L, Philip B, Schiffer-Mannioui C (2015). Multiplex genome-edited T-cell manufacturing platform for “off-the-shelf” adoptive t-cell immunotherapies. Cancer Res.

[10] Kwon YW, Ahn HS, Lee JW (2021). HLA DR genome editing with TALENs in human iPSCs produced immune-tolerant dendritic cells. Stem Cells Int.

[11] Tang Y, Gao L, Feng W (2021). The CRISPR-Cas toolbox for analytical and diagnostic assay development. Chem Soc Rev.

[12] Baglaenko Y, Macfarlane D, Marson A (2021). Genome editing to define the function of risk loci and variants in rheumatic disease. Nat Rev Rheumatol.

[13] Mohanraju P, Makarova KS, Zetsche B (2016). Diverse evolutionary roots and mechanistic variations of the CRISPR-Cas systems. Science.

[14] Lu Y, Xue J, Deng T (2020). Safety and feasibility of CRISPR-edited T cells in patients with refractory non-small-cell lung cancer. Nat Med.

[15] Stadtmauer EA, Fraietta JA, Davis MM (2020). CRISPR-engineered T cells in patients with refractory cancer. Science.

[16] Oft M (2019). Immune regulation and cytotoxic T cell activation of IL-10 agonists - preclinical and clinical experience. Semin Immunol.

[17] Hashimoto M, Im SJ, Araki K (2019). Cytokine-mediated regulation of CD8 T-cell responses during acute and chronic viral infection. Cold Spring Harb Perspect Biol.

[18] Sabharwal SS, Rosen DB, Grein J (2018). GITR agonism enhances cellular metabolism to support CD8(+) T-cell proliferation and effector cytokine production in a mouse tumor model. Cancer Immunol Res.

[19] Lee YJ, Won TJ, Hyung KE (2016). IL-6 induced proliferation and cytotoxic activity of CD8(+) T cells is elevated by SUMO2 overexpression. Arch Pharm Res.

[20] Nowyhed HN, Huynh TR, Thomas GD (2015). Cutting edge: the orphan nuclear receptor Nr4a1 regulates CD8+ T cell expansion and effector function through direct repression of Irf4. J Immunol.

[21] Tschismarov R, Firner S, Gil-Cruz C (2014). HDAC1 controls CD8+ T cell homeostasis and antiviral response. PLoS One.

[22] Ryu MS, Woo MY, Kwon D (2014). Accumulation of cytolytic CD8+ T cells in B16-melanoma and proliferation of mature T cells in TIS21-knockout mice after T cell receptor stimulation. Exp Cell Res.

[23] Feau S, Schoenberger SP, Altman A (2013). SLAT regulates CD8+ T cell clonal expansion in a Cdc42- and NFAT1-dependent manner. J Immunol.

[24] Munitic I, Kuka M, Allam A (2013). CD70 deficiency impairs effector CD8 T cell generation and viral clearance but is dispensable for the recall response to lymphocytic choriomeningitis virus. J Immunol.

[25] Hamilton SE, Jameson SC (2012). CD8 T cell quiescence revisited. Trends Immunol.

[26] Slebioda TJ, Rowley TF, Ferdinand JR (2011). Triggering of TNFRSF25 promotes CD8(+) T-cell responses and anti-tumor immunity. Eur J Immunol.

[27] Randall KL, Chan SS, Ma CS (2011). DOCK8 deficiency impairs CD8 T cell survival and function in humans and mice. J Exp Med.

[28] Schuster K, Gadiot J, Andreesen R (2009). Homeostatic proliferation of naive CD8+ T cells depends on CD62L/L-selectin-mediated homing to peripheral LN. Eur J Immunol.

[29] Bekiaris V, Gaspal F, Kim MY (2009). Synergistic OX40 and CD30 signals sustain CD8+ T cells during antigenic challenge. Eur J Immunol.

[30] Rubinstein MP, Lind NA, Purton JF (2008). IL-7 and IL-15 differentially regulate CD8+ T-cell subsets during contraction of the immune response. Blood.

[31] Ruby CE, Redmond WL, Haley D (2007). Anti-OX40 stimulation in vivo enhances CD8+ memory T cell survival and significantly increases recall responses. Eur J Immunol.

[32] Ueda N, Kuki H, Kamimura D (2006). CD1d-restricted NKT cell activation enhanced homeostatic proliferation of CD8+ T cells in a manner dependent on IL-4. Int Immunol.

[33] Camara NO, Sebille F, Lechler RI (2003). Human CD4+CD25+ regulatory cells have marked and sustained effects on CD8+ T cell activation. Eur J Immunol.

[34] Laderach D, Movassagh M, Johnson A (2002). 4-1BB co-stimulation enhances human CD8(+) T cell priming by augmenting the proliferation and survival of effector CD8(+) T cells. Int Immunol.

[35] Kieper WC, Prlic M, Schmidt CS (2001). Il-12 enhances CD8 T cell homeostatic expansion. J Immunol.

[36] Giri JG, Kumaki S, Ahdieh M (1995). Identification and cloning of a novel IL-15 binding protein that is structurally related to the alpha chain of the IL-2 receptor. EMBO J.

[37] Grabstein KH, Eisenman J, Shanebeck K (1994). Cloning of a T cell growth factor that interacts with the beta chain of the interleukin-2 receptor. Science.

[38] Manna A, Kellett T, Aulakh S (2020). Targeting CD38 is lethal to Breg-like chronic lymphocytic leukemia cells and Tregs, but restores CD8+ T-cell responses. Blood Adv.

[39] Wei H, Geng J, Shi B (2016). Cutting edge: Foxp1 controls naive CD8+ T cell quiescence by simultaneously repressing key pathways in cellular metabolism and cell cycle progression. J Immunol.

[40] Vigano S, Banga R, Bellanger F (2014). CD160-associated CD8 T-cell functional impairment is independent of PD-1 expression. PLoS Pathog.

[41] Chen J, Bruce D, Cantorna MT (2014). Vitamin D receptor expression controls proliferation of naive CD8+ T cells and development of CD8 mediated gastrointestinal inflammation. BMC Immunol.

[42] Kuchroo VK, Anderson AC, Petrovas C (2014). Coinhibitory receptors and CD8 T cell exhaustion in chronic infections. Curr Opin HIV AIDS.

[43] Sullivan JA, Kim EH, Plisch EH (2012). FOXO3 regulates CD8 T cell memory by T cell-intrinsic mechanisms. PLoS Pathog.

[44] Sondergaard H, Coquet JM, Uldrich AP (2009). Endogenous IL-21 restricts CD8+ T cell expansion and is not required for tumor immunity. J Immunol.

[45] Tao J, Gao Y, Li MO (2007). JNK2 negatively regulates CD8+ T cell effector function and anti-tumor immune response. Eur J Immunol.

[46] Biswas PS, Pedicord V, Ploss A (2007). Pathogen-specific CD8 T cell responses are directly inhibited by IL-10. J Immunol.

[47] Khan O, Giles JR, Mcdonald S (2019). TOX transcriptionally and epigenetically programs CD8(+) T cell exhaustion. Nature.

[48] Man K, Gabriel SS, Liao Y (2017). Transcription factor IRF4 promotes CD8(+) T cell exhaustion and limits the development of memory-like T cells during chronic infection. Immunity.

[49] Chen J, Lopez-Moyado IF, Seo H (2019). NR4A transcription factors limit CAR T cell function in solid tumours. Nature.

[50] Martinez GJ, Pereira RM, Aijo T (2015). The transcription factor NFAT promotes exhaustion of activated CD8(+) T cells. Immunity.

[51] Ahmadzadeh M, Johnson LA, Heemskerk B (2009). Tumor antigen-specific CD8 T cells infiltrating the tumor express high levels of PD-1 and are functionally impaired. Blood.

[52] Wolf Y, Anderson AC, Kuchroo VK (2020). TIM3 comes of age as an inhibitory receptor. Nat Rev Immunol.

[53] Guo M, Yuan F, Qi F (2020). Expression and clinical significance of LAG-3, FGL1, PD-L1 and CD8(+)T cells in hepatocellular carcinoma using multiplex quantitative analysis. J Transl Med.

[54] Du X, Tang F, Liu M (2018). A reappraisal of CTLA-4 checkpoint blockade in cancer immunotherapy. Cell Res.

[55] Chauvin JM, Pagliano O, Fourcade J (2015). TIGIT and PD-1 impair tumor antigen-specific CD8(+) T cells in melanoma patients. J Clin Invest.

[56] Joshi NS, Cui W, Chandele A (2007). Inflammation directs memory precursor and short-lived effector CD8(+) T cell fates via the graded expression of T-bet transcription factor. Immunity.

[57] Hwang S, Cobb DA, Bhadra R (2016). Blimp-1-mediated CD4 T cell exhaustion causes CD8 T cell dysfunction during chronic toxoplasmosis. J Exp Med.

[58] Aki D, Li Q, Li H (2019). Immune regulation by protein ubiquitination: roles of the E3 ligases VHL and Itch. Protein Cell.

[59] Delpoux A, Lai CY, Hedrick SM (2017). FOXO1 opposition of CD8(+) T cell effector programming confers early memory properties and phenotypic diversity. Proc Natl Acad Sci U S A.

[60] Feng X, Wang H, Takata H (2011). Transcription factor Foxp1 exerts essential cell-intrinsic regulation of the quiescence of naive T cells. Nat Immunol.

[61] Siddiqui I, Schaeuble K, Chennupati V (2019). Intratumoral Tcf1(+)PD-1(+)CD8(+) T cells with stem-like properties promote tumor control in response to vaccination and checkpoint blockade immunotherapy. Immunity.

[62] Yonesaka K, Haratani K, Takamura S (2018). B7-H3 negatively modulates CTL-mediated cancer immunity. Clin Cancer Res.

[63] Allard B, Pommey S, Smyth MJ (2013). Targeting CD73 enhances the antitumor activity of anti-PD-1 and anti-CTLA-4 mAbs. Clin Cancer Res.

[64] Liu Y, Zhou N, Zhou L (2021). IL-2 regulates tumor-reactive CD8(+) T cell exhaustion by activating the aryl hydrocarbon receptor. Nat Immunol.

[65] Wherry EJ (2011). T cell exhaustion. Nat Immunol.

[66] Henriksson J, Chen X, Gomes T (2019). Genome-wide CRISPR screens in T helper cells reveal pervasive crosstalk between activation and differentiation. Cell.

[67] Cortez JT, Montauti E, Shifrut E (2020). CRISPR screen in regulatory T cells reveals modulators of Foxp3. Nature.

[68] Loo CS, Gatchalian J, Liang Y (2020). A genome-wide CRISPR screen reveals a role for the non-canonical nucleosome-remodeling BAF complex in Foxp3 expression and regulatory T cell function. Immunity.

[69] Spolski R, Li P, Leonard WJ (2018). Biology and regulation of IL-2: from molecular mechanisms to human therapy. Nat Rev Immunol.

[70] Mitchell JL, Seng A, Yankee TM (2016). Expression patterns of Ikaros family members during positive selection and lineage commitment of human thymocytes. Immunology.

[71] Callender LA, Schroth J, Carroll EC (2021). GATA3 induces mitochondrial biogenesis in primary human CD4(+) T cells during DNA damage. Nat Commun.

[72] Ruterbusch M, Pruner KB, Shehata L (2020). In Vivo CD4(+) T cell differentiation and function: revisiting the Th1/Th2 paradigm. Annu Rev Immunol.

[73] Bilate AM, London M, Castro T (2020). T cell receptor is required for differentiation, but not maintenance, of intestinal CD4(+) intraepithelial lymphocytes. Immunity.

[74] Pesce J, Kaviratne M, Ramalingam TR (2006). The IL-21 receptor augments Th2 effector function and alternative macrophage activation. J Clin Invest.

[75] Wurster AL, Rodgers VL, Satoskar AR (2002). Interleukin 21 is a T helper (Th) cell 2 cytokine that specifically inhibits the differentiation of naive Th cells into interferon gamma-producing Th1 cells. J Exp Med.

[76] Saini C, Srivastava RK, Tarique M (2020). Elevated IL-6R on CD4(+) T cells promotes IL-6 driven Th17 cell responses in patients with T1R leprosy reactions. Sci Rep.

[77] Nie J, Zhao Q (2020). Lnc-ITSN1-2, derived from RNA sequencing, correlates with increased disease risk, activity and promotes CD4(+) T cell activation, proliferation and Th1/Th17 cell differentiation by serving as a ceRNA for IL-23R via sponging miR-125a in inflammatory bowel disease. Front Immunol.

[78] Afkarian M, Sedy JR, Yang J (2002). T-bet is a STAT1-induced regulator of IL-12R expression in naive CD4+ T cells. Nat Immunol.

[79] Roessner PM, Llao CL, Lupar E (2021). EOMES and IL-10 regulate antitumor activity of T regulatory type 1 CD4(+) T cells in chronic lymphocytic leukemia. Leukemia.

[80] Balic A, Harcus YM, Taylor MD (2006). IL-4R signaling is required to induce IL-10 for the establishment of T(h)2 dominance. Int Immunol.

[81] Newcomb DC, Zhou W, Moore ML (2009). A functional IL-13 receptor is expressed on polarized murine CD4+ Th17 cells and IL-13 signaling attenuates Th17 cytokine production. J Immunol.

[82] Dolgachev V, Petersen BC, Budelsky AL (2009). Pulmonary IL-17E (IL-25) production and IL-17RB+ myeloid cell-derived Th2 cytokine production are dependent upon stem cell factor-induced responses during chronic allergic pulmonary disease. J Immunol.

[83] Luo CT, Liao W, Dadi S (2016). Graded Foxo1 activity in Treg cells differentiates tumour immunity from spontaneous autoimmunity. Nature.

[84] Xu K, Yang WY, Nanayakkara GK (2018). GATA3, HDAC6, and BCL6 regulate FOXP3+ Treg plasticity and determine Treg conversion into either novel antigen-presenting cell-like Treg or Th1-Treg. Front Immunol.

[85] Alvisi G, Brummelman J, Puccio S (2020). IRF4 instructs effector Treg differentiation and immune suppression in human cancer. J Clin Invest.

[86] Park GY, Lee GW, Kim S (2020). Deletion timing of Cic alleles during hematopoiesis determines the degree of peripheral CD4(+) T cell activation and proliferation. Immune Netw.

[87] Felices M, Lenvik AJ, Mcelmurry R, et al. Continuous treatment with IL-15 exhausts human NK cells via a metabolic defect. JCI Insight. 2018;3(3):e96219. 10.1172/jci.insight.96219 PMC5821201 29415897

[88] Sharma R, Das A (2018). IL-2 mediates NK cell proliferation but not hyperactivity. Immunol Res.

[89] Ivanova DL, Mundhenke TM, Gigley JP (2019). The IL-12- and IL-23-dependent NK cell response is essential for protective immunity against secondary toxoplasma gondii infection. J Immunol.

[90] Davis MR, Zhu Z, Hansen DM (2015). The role of IL-21 in immunity and cancer. Cancer Lett.

[91] Romee R, Schneider SE, Leong JW (2012). Cytokine activation induces human memory-like NK cells. Blood.

[92] Pahl J, Koch J, Gotz JJ (2018). CD16A activation of NK cells promotes NK cell proliferation and memory-like cytotoxicity against cancer cells. Cancer Immunol Res.

[93] Holder KA, Grant MD (2019). Human cytomegalovirus IL-10 augments NK cell cytotoxicity. J Leukoc Biol.

[94] Rabacal W, Pabbisetty SK, Hoek KL (2016). Transcription factor KLF2 regulates homeostatic NK cell proliferation and survival. Proc Natl Acad Sci U S A.

[95] Kweon S, Phan MT, Chun S (2019). Expansion of human NK cells using K562 cells expressing OX40 ligand and short exposure to IL-21. Front Immunol.

[96] Braun M, Bjorkstrom NK, Gupta S (2014). NK cell activation in human hantavirus infection explained by virus-induced IL-15/IL15Ralpha expression. PLoS Pathog.

[97] Hayakawa K, Salmeron MA, Kornbluth J (1991). The role of IL-4 in proliferation and differentiation of human natural killer cells. Study of an IL-4-dependent versus an IL-2-dependent natural killer cell clone. J Immunol.

[98] Ida H, Anderson P (1998). Activation-induced NK cell death triggered by CD2 stimulation. Eur J Immunol.

[99] Gonzalez C, Chames P, Kerfelec B (2019). Nanobody-CD16 catch bond reveals NK cell mechanosensitivity. Biophys J.

[100] Mccarthy MT, Lin D, Soga T (2020). Inosine pranobex enhances human NK cell cytotoxicity by inducing metabolic activation and NKG2D ligand expression. Eur J Immunol.

[101] Imai C, Iwamoto S, Campana D (2005). Genetic modification of primary natural killer cells overcomes inhibitory signals and induces specific killing of leukemic cells. Blood.

[102] Galazka G, Domowicz M, Ewiak-Paszynska A (2019). NK cell induced T cell anergy depends on GRAIL expression. Cells.

[103] Stark S, Watzl C (2006). 2B4 (CD244), NTB-A and CRACC (CS1) stimulate cytotoxicity but no proliferation in human NK cells. Int Immunol.

[104] Zhang J, Sun R, Wei H (2004). Characterization of stem cell factor gene-modified human natural killer cell line, NK-92 cells: implication in NK cell-based adoptive cellular immunotherapy. Oncol Rep.

[105] Zhang Q, Bi J, Zheng X (2018). Blockade of the checkpoint receptor TIGIT prevents NK cell exhaustion and elicits potent anti-tumor immunity. Nat Immunol.

[106] Khan M, Arooj S, Wang H (2020). NK cell-based immune checkpoint inhibition. Front Immunol.

[107] Zhang C, Liu Y (2020). Targeting NK cell checkpoint receptors or molecules for cancer immunotherapy. Front Immunol.

[108] Muller-Durovic B, Lanna A, Covre LP (2016). Killer cell lectin-like receptor G1 inhibits NK cell function through activation of adenosine 5′-monophosphate-activated protein kinase. J Immunol.

[109] Bottino C, Castriconi R, Pende D (2003). Identification of PVR (CD155) and Nectin-2 (CD112) as cell surface ligands for the human DNAM-1 (CD226) activating molecule. J Exp Med.

[110] Hawinkels LJ, Paauwe M, Verspaget HW (2014). Interaction with colon cancer cells hyperactivates TGF-β signaling in cancer-associated fibroblasts. Oncogene.

[111] Sundaram B, Behnke K, Belancic A (2019). iRhom2 inhibits bile duct obstruction-induced liver fibrosis. Sci Signal.

[112] Yauch RL, Gould SE, Scales SJ (2008). A paracrine requirement for hedgehog signalling in cancer. Nature.

[113] Ferrer-Mayorga G, Gómez-López G, Barbáchano A (2017). Vitamin D receptor expression and associated gene signature in tumour stromal fibroblasts predict clinical outcome in colorectal cancer. Gut.

[114] Erez N, Truitt M, Olson P (2010). Cancer-associated fibroblasts are activated in incipient neoplasia to orchestrate tumor-promoting inflammation in an NF-kappaB-dependent manner. Cancer Cell.

[115] Sanz-Moreno V, Gaggioli C, Yeo M (2011). ROCK and JAK1 signaling cooperate to control actomyosin contractility in tumor cells and stroma. Cancer Cell.

[116] Ershaid N, Sharon Y, Doron H (2019). NLRP3 inflammasome in fibroblasts links tissue damage with inflammation in breast cancer progression and metastasis. Nat Commun.

[117] Ferrari N, Ranftl R, Chicherova I (2019). Dickkopf-3 links HSF1 and YAP/TAZ signalling to control aggressive behaviours in cancer-associated fibroblasts. Nat Commun.

[118] Fu L, Zhang C, Zhang LY (2011). Wnt2 secreted by tumour fibroblasts promotes tumour progression in oesophageal cancer by activation of the Wnt/β-catenin signalling pathway. Gut.

[119] Orimo A, Gupta PB, Sgroi DC (2005). Stromal fibroblasts present in invasive human breast carcinomas promote tumor growth and angiogenesis through elevated SDF-1/CXCL12 secretion. Cell.

[120] Wu MH, Hong HC, Hong TM (2011). Targeting galectin-1 in carcinoma-associated fibroblasts inhibits oral squamous cell carcinoma metastasis by downregulating MCP-1/CCL2 expression. Clin Cancer Res.

[121] Tsuyada A, Chow A, Wu J (2012). CCL2 mediates cross-talk between cancer cells and stromal fibroblasts that regulates breast cancer stem cells. Cancer Res.

[122] Liu J, Chen S, Wang W (2016). Cancer-associated fibroblasts promote hepatocellular carcinoma metastasis through chemokine-activated hedgehog and TGF-β pathways. Cancer Lett.

[123] Mishra P, Banerjee D, Ben-Baruch A (2011). Chemokines at the crossroads of tumor-fibroblast interactions that promote malignancy. J Leukoc Biol.

[124] Zhou Q, Wu X, Wang X (2020). The reciprocal interaction between tumor cells and activated fibroblasts mediated by TNF-α/IL-33/ST2L signaling promotes gastric cancer metastasis. Oncogene.

[125] Ford K, Hanley CJ, Mellone M (2020). NOX4 inhibition potentiates immunotherapy by overcoming cancer-associated fibroblast-mediated CD8 T-cell exclusion from tumors. Cancer Res.

[126] Kuen J, Darowski D, Kluge T (2017). Pancreatic cancer cell/fibroblast co-culture induces M2 like macrophages that influence therapeutic response in a 3D model. PLoS One.

[127] Gorchs L, Fernández MC, Bankhead P (2019). Human pancreatic carcinoma-associated fibroblasts promote expression of co-inhibitory markers on CD4(+) and CD8(+) T-cells. Front Immunol.

[128] Lau EY, Lo J, Cheng BY (2016). Cancer-associated fibroblasts regulate tumor-initiating cell plasticity in hepatocellular carcinoma through c-Met/FRA1/HEY1 signaling. Cell Rep.

[129] Yu B, Wu K, Wang X (2018). Periostin secreted by cancer-associated fibroblasts promotes cancer stemness in head and neck cancer by activating protein tyrosine kinase 7. Cell Death Dis.

[130] Lenos KJ, Miedema DM, Lodestijn SC (2018). Stem cell functionality is microenvironmentally defined during tumour expansion and therapy response in colon cancer. Nat Cell Biol.

[131] Locatelli A, Lofgren KA, Daniel AR (2012). Mechanisms of HGF/Met signaling to Brk and Sam68 in breast cancer progression. Horm Cancer.

[132] Henriksson ML, Edin S, Dahlin AM (2011). Colorectal cancer cells activate adjacent fibroblasts resulting in FGF1/FGFR3 signaling and increased invasion. Am J Pathol.

[133] Baeriswyl V, Christofori G (2009). The angiogenic switch in carcinogenesis. Semin Cancer Biol.

[134] Shimo T, Kubota S, Kondo S (2001). Connective tissue growth factor as a major angiogenic agent that is induced by hypoxia in a human breast cancer cell line. Cancer Lett.

[135] Saito RA, Micke P, Paulsson J (2010). Forkhead box F1 regulates tumor-promoting properties of cancer-associated fibroblasts in lung cancer. Cancer Res.

[136] Lotti F, Jarrar AM, Pai RK (2013). Chemotherapy activates cancer-associated fibroblasts to maintain colorectal cancer-initiating cells by IL-17A. J Exp Med.

[137] Goetz JG, Minguet S, Navarro-Lérida I (2011). Biomechanical remodeling of the microenvironment by stromal caveolin-1 favors tumor invasion and metastasis. Cell.

[138] Lee HO, Mullins SR, Franco-Barraza J (2011). FAP-overexpressing fibroblasts produce an extracellular matrix that enhances invasive velocity and directionality of pancreatic cancer cells. BMC Cancer.

[139] Sun L, Wang Y, Wang L (2019). Resolvin D1 prevents epithelial-mesenchymal transition and reduces the stemness features of hepatocellular carcinoma by inhibiting paracrine of cancer-associated fibroblast-derived COMP. J Exp Clin Cancer Res.

[140] Hu M, Peluffo G, Chen H (2009). Role of COX-2 in epithelial-stromal cell interactions and progression of ductal carcinoma in situ of the breast. Proc Natl Acad Sci U S A.

[141] Meierjohann S, Hufnagel A, Wende E (2010). MMP13 mediates cell cycle progression in melanocytes and melanoma cells: in vitro studies of migration and proliferation. Mol Cancer.

[142] Leung CS, Yeung TL, Yip KP (2014). Calcium-dependent FAK/CREB/TNNC1 signalling mediates the effect of stromal MFAP5 on ovarian cancer metastatic potential. Nat Commun.

[143] Mizutani Y, Kobayashi H, Iida T (2019). Meflin-positive cancer-associated fibroblasts inhibit pancreatic carcinogenesis. Cancer Res.

[144] Madar S, Brosh R, Buganim Y (2009). Modulated expression of WFDC1 during carcinogenesis and cellular senescence. Carcinogenesis.

[145] Banerjee S, Halder K, Bose A (2011). TLR signaling-mediated differential histone modification at IL-10 and IL-12 promoter region leads to functional impairments in tumor-associated macrophages. Carcinogenesis.

[146] Mantovani A, Allavena P (2015). The interaction of anticancer therapies with tumor-associated macrophages. J Exp Med.

[147] Wang YC, He F, Feng F (2010). Notch signaling determines the M1 versus M2 polarization of macrophages in antitumor immune responses. Cancer Res.

[148] Giurisato E, Xu Q, Lonardi S (2018). Myeloid ERK5 deficiency suppresses tumor growth by blocking protumor macrophage polarization via STAT3 inhibition. Proc Natl Acad Sci U S A.

[149] Xiang W, Shi R, Kang X (2018). Monoacylglycerol lipase regulates cannabinoid receptor 2-dependent macrophage activation and cancer progression. Nat Commun.

[150] Petrillo M, Zannoni GF, Martinelli E (2015). Polarisation of tumor-associated macrophages toward M2 phenotype correlates with poor response to chemoradiation and reduced survival in patients with locally advanced cervical cancer. PLoS One.

[151] Jeyakumar T, Fodil N, Van Der Kraak L (2019). Inactivation of interferon regulatory factor 1 causes susceptibility to colitis-associated colorectal cancer. Sci Rep.

[152] Digiacomo G, Ziche M, Dello SP (2015). Prostaglandin E2 transactivates the colony-stimulating factor-1 receptor and synergizes with colony-stimulating factor-1 in the induction of macrophage migration via the mitogen-activated protein kinase ERK1/2. FASEB J.

[153] Zhao P, Gao D, Wang Q (2015). Response gene to complement 32 (RGC-32) expression on M2-polarized and tumor-associated macrophages is M-CSF-dependent and enhanced by tumor-derived IL-4. Cell Mol Immunol.

[154] Lanaya H, Natarajan A, Komposch K (2014). EGFR has a tumour-promoting role in liver macrophages during hepatocellular carcinoma formation. Nat Cell Biol.

[155] Ambade A, Satishchandran A, Saha B (2016). Hepatocellular carcinoma is accelerated by NASH involving M2 macrophage polarization mediated by hif-1αinduced IL-10. Oncoimmunology.

[156] Chen P, Zuo H, Xiong H (2017). Gpr132 sensing of lactate mediates tumor-macrophage interplay to promote breast cancer metastasis. Proc Natl Acad Sci U S A.

[157] Lin Y, Xu J, Lan H (2019). Tumor-associated macrophages in tumor metastasis: biological roles and clinical therapeutic applications. J Hematol Oncol.

[158] Satoh T, Takeuchi O, Vandenbon A (2010). The Jmjd3-Irf4 axis regulates M2 macrophage polarization and host responses against helminth infection. Nat Immunol.

[159] Nakanishi Y, Nakatsuji M, Seno H (2011). COX-2 inhibition alters the phenotype of tumor-associated macrophages from M2 to M1 in ApcMin/+ mouse polyps. Carcinogenesis.

[160] Li C, Xu X, Wei S, et al. Tumor-associated macrophages: potential therapeutic strategies and future prospects in cancer. J Immunother Cancer. 2021;9(1):e001341. 10.1136/jitc-2020-001341 PMC8728363 33504575

[161] Hammerl D, Massink M, Smid M (2020). Clonality, antigen recognition, and suppression of CD8(+) T cells differentially affect prognosis of breast cancer subtypes. Clin Cancer Res.

[162] Hurkmans DP, Kuipers ME, Smit J (2020). Tumor mutational load, CD8(+) T cells, expression of PD-L1 and HLA class I to guide immunotherapy decisions in NSCLC patients. Cancer Immunol Immunother.

[163] Crespo J, Sun H, Welling TH (2013). T cell anergy, exhaustion, senescence, and stemness in the tumor microenvironment. Curr Opin Immunol.

[164] Morgan DA, Ruscetti FW, Gallo R (1976). Selective in vitro growth of T lymphocytes from normal human bone marrows. Science.

[165] Atkins MB, Lotze MT, Dutcher JP (1999). High-dose recombinant interleukin 2 therapy for patients with metastatic melanoma: analysis of 270 patients treated between 1985 and 1993. J Clin Oncol.

[166] Rosenberg SA, Yang JC, Topalian SL (1994). Treatment of 283 consecutive patients with metastatic melanoma or renal cell cancer using high-dose bolus interleukin 2. JAMA.

[167] Ahmadzadeh M, Rosenberg SA (2006). IL-2 administration increases CD4+ CD25(hi) Foxp3+ regulatory T cells in cancer patients. Blood.

[168] Sim GC, Martin-Orozco N, Jin L (2014). IL-2 therapy promotes suppressive ICOS+ Treg expansion in melanoma patients. J Clin Invest.

[169] Sockolosky JT, Trotta E, Parisi G (2018). Selective targeting of engineered T cells using orthogonal IL-2 cytokine-receptor complexes. Science.

[170] Ishihara J, Ishihara A, Sasaki K (2019). Targeted antibody and cytokine cancer immunotherapies through collagen affinity. Sci Transl Med.

[171] Perna SK, Pagliara D, Mahendravada A (2014). Interleukin-7 mediates selective expansion of tumor-redirected cytotoxic T lymphocytes (CTLs) without enhancement of regulatory T-cell inhibition. Clin Cancer Res.

[172] Gong W, Hoffmann JM, Stock S (2019). Comparison of IL-2 vs IL-7/IL-15 for the generation of NY-ESO-1-specific T cells. Cancer Immunol Immunother.

[173] Santegoets SJ, Turksma AW, Suhoski MM (2013). IL-21 promotes the expansion of CD27+ CD28+ tumor infiltrating lymphocytes with high cytotoxic potential and low collateral expansion of regulatory T cells. J Transl Med.

[174] Tirosh I, Izar B, Prakadan SM (2016). Dissecting the multicellular ecosystem of metastatic melanoma by single-cell RNA-seq. Science.

[175] Seo H, Gonzalez-Avalos E, Zhang W (2021). BATF and IRF4 cooperate to counter exhaustion in tumor-infiltrating CAR T cells. Nat Immunol.

[176] Seo H, Chen J, Gonzalez-Avalos E (2019). TOX and TOX2 transcription factors cooperate with NR4A transcription factors to impose CD8(+) T cell exhaustion. Proc Natl Acad Sci U S A.

[177] Kurachi M (2019). CD8(+) T cell exhaustion. Semin Immunopathol.

[178] Scott AC, Dundar F, Zumbo P (2019). TOX is a critical regulator of tumour-specific T cell differentiation. Nature.

[179] Han HS, Jeong S, Kim H (2021). TOX-expressing terminally exhausted tumor-infiltrating CD8(+) T cells are reinvigorated by co-blockade of PD-1 and TIGIT in bladder cancer. Cancer Lett.

[180] Wang X, He Q, Shen H (2019). TOX promotes the exhaustion of antitumor CD8(+) T cells by preventing PD1 degradation in hepatocellular carcinoma. J Hepatol.

[181] Marotte L, Simon S, Vignard V, et al. Increased antitumor efficacy of PD-1-deficient melanoma-specific human lymphocytes. J Immunother Cancer. 2020;8(1):e000311. 10.1136/jitc-2019-000311 PMC7057432 32001504

[182] Menger L, Sledzinska A, Bergerhoff K (2016). TALEN-mediated inactivation of PD-1 in tumor-reactive lymphocytes promotes intratumoral T-cell persistence and rejection of established tumors. Cancer Res.

[183] Nakazawa T, Natsume A, Nishimura F (2020). Effect of CRISPR/Cas9-mediated PD-1-disrupted primary human third-generation CAR-T cells targeting EGFRvIII on in vitro human glioblastoma cell growth. Cells.

[184] Rafiq S, Yeku OO, Jackson HJ (2018). Targeted delivery of a PD-1-blocking scFv by CAR-T cells enhances anti-tumor efficacy in vivo. Nat Biotechnol.

[185] Chen X, Yang S, Li S (2021). Secretion of bispecific protein of anti-PD-1 fused with TGF-beta trap enhances antitumor efficacy of CAR-T cell therapy. Mol Ther Oncolytics.

[186] Blaeschke F, Stenger D, Apfelbeck A (2021). Augmenting anti-CD19 and anti-CD22 CAR T-cell function using PD-1-CD28 checkpoint fusion proteins. Blood Cancer J.

[187] Liang Y, Liu H, Lu Z (2021). CD19 CAR-T expressing PD-1/CD28 chimeric switch receptor as a salvage therapy for DLBCL patients treated with different CD19-directed CAR T-cell therapies. J Hematol Oncol.

[188] Liu H, Lei W, Zhang C (2021). CD19-specific CAR T Cells that Express a PD-1/CD28 Chimeric Switch-Receptor are Effective in Patients with PD-L1-positive B-Cell Lymphoma. Clin Cancer Res.

[189] Wang Z, Li N, Feng K (2021). Phase I study of CAR-T cells with PD-1 and TCR disruption in mesothelin-positive solid tumors. Cell Mol Immunol.

[190] Esensten JH, Helou YA, Chopra G (2016). CD28 costimulation: from mechanism to therapy. Immunity.

[191] Turtle CJ, Hanafi LA, Berger C (2016). CD19 CAR-T cells of defined CD4+:CD8+ composition in adult B cell ALL patients. J Clin Invest.

[192] Borst J, Ahrends T, Babala N (2018). CD4(+) T cell help in cancer immunology and immunotherapy. Nat Rev Immunol.

[193] Kunicki MA, Amaya HL, Davis KL (2018). Identity and diversity of human peripheral Th and T regulatory cells defined by single-cell mass cytometry. J Immunol.

[194] Ng M, Roth TL, Mendoza VF (2019). Helios enhances the preferential differentiation of human fetal CD4(+) naive T cells into regulatory T cells. Sci Immunol.

[195] Simeonov DR, Gowen BG, Boontanrart M (2017). Discovery of stimulation-responsive immune enhancers with CRISPR activation. Nature.

[196] Li C, Jiang P, Wei S (2020). Regulatory T cells in tumor microenvironment: new mechanisms, potential therapeutic strategies and future prospects. Mol Cancer.

[197] Overacre-Delgoffe AE, Vignali D (2018). Treg fragility: a prerequisite for effective antitumor immunity?. Cancer Immunol Res.

[198] Rudra D, Deroos P, Chaudhry A (2012). Transcription factor Foxp3 and its protein partners form a complex regulatory network. Nat Immunol.

[199] Schumann K, Raju SS, Lauber M (2020). Functional CRISPR dissection of gene networks controlling human regulatory T cell identity. Nat Immunol.

[200] Chou CK, Turtle CJ (2019). Insight into mechanisms associated with cytokine release syndrome and neurotoxicity after CD19 CAR-T cell immunotherapy. Bone Marrow Transplant.

[201] Liu E, Marin D, Banerjee P (2020). Use of CAR-transduced natural killer cells in CD19-positive lymphoid tumors. N Engl J Med.

[202] Hunter BD, Jacobson CA (2019). CAR T-cell associated neurotoxicity: mechanisms, clinicopathologic correlates, and future directions. J Natl Cancer Inst.

[203] Shimasaki N, Jain A, Campana D (2020). NK cells for cancer immunotherapy. Nat Rev Drug Discov.

[204] Xu X, Huang W, Heczey A (2019). NKT cells coexpressing a GD2-specific chimeric antigen receptor and IL15 show enhanced in vivo persistence and antitumor activity against neuroblastoma. Clin Cancer Res.

[205] Imamura M, Shook D, Kamiya T (2014). Autonomous growth and increased cytotoxicity of natural killer cells expressing membrane-bound interleukin-15. Blood.

[206] Lin C, Zhang J (2018). Reformation in chimeric antigen receptor based cancer immunotherapy: redirecting natural killer cell. Biochim Biophys Acta Rev Cancer.

[207] Xie G, Dong H, Liang Y (2020). CAR-NK cells: a promising cellular immunotherapy for cancer. EBioMedicine.

[208] Huang RS, Shih HA, Lai MC (2020). Enhanced NK-92 cytotoxicity by CRISPR genome engineering using Cas9 ribonucleoproteins. Front Immunol.

[209] Wu SY, Fu T, Jiang YZ (2020). Natural killer cells in cancer biology and therapy. Mol Cancer.

[210] Park SM, Do-Thi VA, Lee JO (2020). Interleukin-9 inhibits lung metastasis of melanoma through stimulating anti-tumor M1 macrophages. Mol Cells.

[211] Chiba Y, Mizoguchi I, Furusawa J (2018). Interleukin-27 exerts its antitumor effects by promoting differentiation of hematopoietic stem cells to M1 macrophages. Cancer Res.

[212] Qiu N, Wang G, Wang J (2021). Tumor-associated macrophage and tumor-cell dually transfecting polyplexes for efficient interleukin-12 cancer gene therapy. Adv Mater.

[213] Hagemann T, Lawrence T, Mcneish I (2008). “Re-educating” tumor-associated macrophages by targeting NF-kappaB. J Exp Med.

[214] Kortylewski M, Xin H, Kujawski M (2009). Regulation of the IL-23 and IL-12 balance by Stat3 signaling in the tumor microenvironment. Cancer Cell.

[215] Qian Y, Qiao S, Dai Y (2017). Molecular-targeted immunotherapeutic strategy for melanoma via dual-targeting nanoparticles delivering small interfering RNA to tumor-associated macrophages. ACS Nano.

[216] Cannarile MA, Weisser M, Jacob W (2017). Colony-stimulating factor 1 receptor (CSF1R) inhibitors in cancer therapy. J Immunother Cancer.

[217] Li X, Yao W, Yuan Y (2017). Targeting of tumour-infiltrating macrophages via CCL2/CCR2 signalling as a therapeutic strategy against hepatocellular carcinoma. Gut.

[218] Yang H, Zhang Q, Xu M (2020). CCL2-CCR2 axis recruits tumor associated macrophages to induce immune evasion through PD-1 signaling in esophageal carcinogenesis. Mol Cancer.

[219] Schmall A, Al-Tamari HM, Herold S (2015). Macrophage and cancer cell cross-talk via CCR2 and CX3CR1 is a fundamental mechanism driving lung cancer. Am J Respir Crit Care Med.

[220] Bartoschek M, Oskolkov N, Bocci M (2018). Spatially and functionally distinct subclasses of breast cancer-associated fibroblasts revealed by single cell RNA sequencing. Nat Commun.

[221] Zhang M, Yang H, Wan L (2020). Single-cell transcriptomic architecture and intercellular crosstalk of human intrahepatic cholangiocarcinoma. J Hepatol.

[222] Helms E, Onate MK, Sherman MH (2020). Fibroblast heterogeneity in the pancreatic tumor microenvironment. Cancer Discov.

[223] Kalluri R (2016). The biology and function of fibroblasts in cancer. Nat Rev Cancer.

[224] Biffi G, Oni TE, Spielman B (2019). IL1-induced JAK/STAT signaling is antagonized by TGFbeta to shape CAF heterogeneity in pancreatic ductal adenocarcinoma. Cancer Discov.

[225] Pein M, Insua-Rodriguez J, Hongu T (2020). Metastasis-initiating cells induce and exploit a fibroblast niche to fuel malignant colonization of the lungs. Nat Commun.

[226] Guillen DN, Sanz-Pamplona R, Berdiel-Acer M (2019). Noncanonical TGFbeta pathway relieves the blockade of IL1beta/TGFbeta-mediated crosstalk between tumor and stroma: TGFBR1 and TAK1 inhibition in colorectal cancer. Clin Cancer Res.

[227] Simpkins SA, Hanby AM, Holliday DL (2012). Clinical and functional significance of loss of caveolin-1 expression in breast cancer-associated fibroblasts. J Pathol.

[228] Hartweger H, Mcguire AT, Horning M (2019). HIV-specific humoral immune responses by CRISPR/Cas9-edited B cells. J Exp Med.

[229] Nahmad AD, Raviv Y, Horovitz-Fried M (2020). Engineered B cells expressing an anti-HIV antibody enable memory retention, isotype switching and clonal expansion. Nat Commun.

[230] Zhang Y, Shen S, Zhao G (2019). In situ repurposing of dendritic cells with CRISPR/Cas9-based nanomedicine to induce transplant tolerance. Biomaterials.

[231] Mao X, Xu J, Wang W (2021). Crosstalk between cancer-associated fibroblasts and immune cells in the tumor microenvironment: new findings and future perspectives. Mol Cancer.

[232] Schuster SJ, Tam CS, Borchmann P (2021). Long-term clinical outcomes of tisagenlecleucel in patients with relapsed or refractory aggressive B-cell lymphomas (JULIET): a multicentre, open-label, single-arm, phase 2 study. Lancet Oncol.

[233] Abramson JS, Palomba ML, Gordon LI (2020). Lisocabtagene maraleucel for patients with relapsed or refractory large B-cell lymphomas (TRANSCEND NHL 001): a multicentre seamless design study. Lancet.

[234] Jiang VC, Liu Y, Jordan A (2021). The antibody drug conjugate VLS-101 targeting ROR1 is effective in CAR T-resistant mantle cell lymphoma. J Hematol Oncol.

[235] Berdeja JG, Madduri D, Usmani SZ (2021). Ciltacabtagene autoleucel, a B-cell maturation antigen-directed chimeric antigen receptor T-cell therapy in patients with relapsed or refractory multiple myeloma (CARTITUDE-1): a phase 1b/2 open-label study. Lancet.

[236] Hou AJ, Chen LC, Chen YY (2021). Navigating CAR-T cells through the solid-tumour microenvironment. Nat Rev Drug Discov.

[237] Hanssens H, Meeus F, De Veirman K, et al. The antigen-binding moiety in the driver's seat of CARs. Med Res Rev. 2022;42(1):306-42. 10.1002/med.21818 PMC9292017 34028069

[238] Maude SL, Frey N, Shaw PA (2014). Chimeric antigen receptor T cells for sustained remissions in leukemia. N Engl J Med.

[239] Kochenderfer JN, Wilson WH, Janik JE (2010). Eradication of B-lineage cells and regression of lymphoma in a patient treated with autologous T cells genetically engineered to recognize CD19. Blood.

[240] Fry TJ, Shah NN, Orentas RJ (2018). CD22-targeted CAR T cells induce remission in B-ALL that is naive or resistant to CD19-targeted CAR immunotherapy. Nat Med.

[241] Zanetti SR, Velasco-Hernandez T, Gutierrez-Aguera F, et al. A novel and efficient tandem CD19- and CD22-directed CAR for B cell ALL. Mol Ther. 2022;30(2):550-63. 10.1016/j.ymthe.2021.08.033 PMC8821938 34478871

[242] Donovan LK, Delaidelli A, Joseph SK (2020). Locoregional delivery of CAR T cells to the cerebrospinal fluid for treatment of metastatic medulloblastoma and ependymoma. Nat Med.

[243] Bielamowicz K, Fousek K, Byrd TT (2018). Trivalent CAR T cells overcome interpatient antigenic variability in glioblastoma. Neuro-Oncology.

[244] Dai H, Zhang W, Li X (2015). Tolerance and efficacy of autologous or donor-derived T cells expressing CD19 chimeric antigen receptors in adult B-ALL with extramedullary leukemia. Oncoimmunology.

[245] Cai B, Guo M, Wang Y (2016). Co-infusion of haplo-identical CD19-chimeric antigen receptor T cells and stem cells achieved full donor engraftment in refractory acute lymphoblastic leukemia. J Hematol Oncol.

[246] Yan Z, Cao J, Cheng H (2019). A combination of humanised anti-CD19 and anti-BCMA CAR T cells in patients with relapsed or refractory multiple myeloma: a single-arm, phase 2 trial. Lancet Haematol.

[247] Tong C, Zhang Y, Liu Y (2020). Optimized tandem CD19/CD20 CAR-engineered T cells in refractory/relapsed B-cell lymphoma. Blood.

[248] Shah NN, Johnson BD, Schneider D (2020). Bispecific anti-CD20, anti-CD19 CAR T cells for relapsed B cell malignancies: a phase 1 dose escalation and expansion trial. Nat Med.

[249] Reed B, Butelman ER, Fry RS (2018). Repeated administration of opra kappa (LY2456302), a novel, short-acting, selective KOP-r antagonist, in persons with and without cocaine dependence. Neuropsychopharmacology.

[250] Wang Y, Zhang WY, Han QW (2014). Effective response and delayed toxicities of refractory advanced diffuse large B-cell lymphoma treated by CD20-directed chimeric antigen receptor-modified T cells. Clin Immunol.

[251] Zhang W, Yang J, Zhou C (2020). Early response observed in pediatric patients with relapsed/refractory Burkitt lymphoma treated with chimeric antigen receptor T cells. Blood.

[252] Pan J, Niu Q, Deng B (2019). CD22 CAR T-cell therapy in refractory or relapsed B acute lymphoblastic leukemia. Leukemia.

[253] Wang CM, Wu ZQ, Wang Y (2017). Autologous T cells expressing CD30 chimeric antigen receptors for relapsed or refractory hodgkin lymphoma: an open-label phase I trial. Clin Cancer Res.

[254] Wang QS, Wang Y, Lv HY (2015). Treatment of CD33-directed chimeric antigen receptor-modified T cells in one patient with relapsed and refractory acute myeloid leukemia. Mol Ther.

[255] Jiang C, Zhao W, Qin M (2019). CD56-chimeric antigen receptor T-cell therapy for refractory/recurrent rhabdomyosarcoma: a 3.5-year follow-up case report. Medicine (Baltimore).

[256] Ji F, Zhang F, Zhang M (2021). Targeting the DNA damage response enhances CD70 CAR-T cell therapy for renal carcinoma by activating the cGAS-STING pathway. J Hematol Oncol.

[257] Yang M, Tang X, Zhang Z (2020). Tandem CAR-T cells targeting CD70 and B7-H3 exhibit potent preclinical activity against multiple solid tumors. Theranostics.

[258] Deng W, Chen P, Lei W (2021). CD70-targeting CAR-T cells have potential activity against CD19-negative B-cell Lymphoma. Cancer Commun (Lond).

[259] Feng KC, Guo YL, Liu Y (2017). Cocktail treatment with EGFR-specific and CD133-specific chimeric antigen receptor-modified T cells in a patient with advanced cholangiocarcinoma. J Hematol Oncol.

[260] Wang Y, Chen M, Wu Z (2018). CD133-directed CAR T cells for advanced metastasis malignancies: a phase I trial. Oncoimmunology.

[261] Tian C, Yang H, Zhu L (2017). Anti-CD138 chimeric antigen receptor-modified T cell therapy for multiple myeloma with extensive extramedullary involvement. Ann Hematol.

[262] Künkele A, Taraseviciute A, Finn LS (2017). Preclinical assessment of CD171-directed CAR T-cell adoptive therapy for childhood neuroblastoma: CE7 epitope target safety and product manufacturing feasibility. Clin Cancer Res.

[263] Feng K, Liu Y, Guo Y (2018). Phase I study of chimeric antigen receptor modified T cells in treating HER2-positive advanced biliary tract cancers and pancreatic cancers. Protein Cell.

[264] Feng K, Guo Y, Dai H (2016). Chimeric antigen receptor-modified T cells for the immunotherapy of patients with EGFR-expressing advanced relapsed/refractory non-small cell lung cancer. Sci China Life Sci.

[265] Guo Y, Feng K, Liu Y (2018). Phase I study of chimeric antigen receptor-modified T cells in patients with EGFR-positive advanced biliary tract cancers. Clin Cancer Res.

[266] Liu Y, Guo Y, Wu Z (2020). Anti-EGFR chimeric antigen receptor-modified T cells in metastatic pancreatic carcinoma: a phase I clinical trial. Cytotherapy.

[267] Lv J, Zhao R, Wu D (2019). Mesothelin is a target of chimeric antigen receptor T cells for treating gastric cancer. J Hematol Oncol.

[268] Ko AH, Jordan AC, Tooker E (2020). Dual targeting of mesothelin and CD19 with chimeric antigen receptor-modified T cells in patients with metastatic pancreatic cancer. Mol Ther.

[269] Haas AR, Tanyi JL, O'Hara MH (2019). Phase I study of lentiviral-transduced chimeric antigen receptor-modified T cells recognizing mesothelin in advanced solid cancers. Mol Ther.

[270] Zhang E, Yang P, Gu J (2018). Recombination of a dual-CAR-modified T lymphocyte to accurately eliminate pancreatic malignancy. J Hematol Oncol.

[271] Harada S, Ando M, Ando J, et al. Dual-antigen targeted iPSC-derived chimeric antigen receptor-T cell therapy for refractory lymphoma. Mol Ther. 2022;30(2):534-49. 10.1016/j.ymthe.2021.10.006 PMC8821952 34628050

[272] Tang X, Tang Q, Mao Y (2019). CD137 co-stimulation improves the antitumor effect of LMP1-specific chimeric antigen receptor T cells in vitro and in vivo. Onco Targets Ther.

[273] Wing A, Fajardo CA, Posey AJ (2018). Improving CART-cell therapy of solid tumors with oncolytic virus-driven production of a bispecific T-cell engager. Cancer Immunol Res.

[274] O'Rourke DM, Nasrallah MP, Desai A (2017). A single dose of peripherally infused EGFRvIII-directed CAR T cells mediates antigen loss and induces adaptive resistance in patients with recurrent glioblastoma. Sci Transl Med.

[275] Choi BD, Yu X, Castano AP (2019). CAR-T cells secreting BiTEs circumvent antigen escape without detectable toxicity. Nat Biotechnol.

[276] Ma W, Zhu D, Li J (2020). Coating biomimetic nanoparticles with chimeric antigen receptor T cell-membrane provides high specificity for hepatocellular carcinoma photothermal therapy treatment. Theranostics.

[277] Pang N, Shi J, Qin L (2021). IL-7 and CCL19-secreting CAR-T cell therapy for tumors with positive glypican-3 or mesothelin. J Hematol Oncol.

[278] Wang X, Cabrera FG, Sharp KL (2021). Engineering tolerance toward allogeneic CAR-T cells by regulation of MHC surface expression with human herpes virus-8 proteins. Mol Ther.

[279] Wu D, Lv J, Zhao R (2020). PSCA is a target of chimeric antigen receptor T cells in gastric cancer. Biomark Res.

[280] Qin L, Lai Y, Zhao R (2017). Incorporation of a hinge domain improves the expansion of chimeric antigen receptor T cells. J Hematol Oncol.

[281] You F, Jiang L, Zhang B (2016). Phase 1 clinical trial demonstrated that MUC1 positive metastatic seminal vesicle cancer can be effectively eradicated by modified Anti-MUC1 chimeric antigen receptor transduced T cells. Sci China Life Sci.

[282] Mao Y, Fan W, Hu H (2019). MAGE-A1 in lung adenocarcinoma as a promising target of chimeric antigen receptor T cells. J Hematol Oncol.

[283] Srivastava S, Salter AI, Liggitt D (2019). Logic-gated ROR1 chimeric antigen receptor expression rescues T cell-mediated toxicity to normal tissues and enables selective tumor targeting. Cancer Cell.

[284] Balakrishnan A, Rajan A, Salter AI (2019). Multispecific targeting with synthetic ankyrin repeat motif chimeric antigen receptors. Clin Cancer Res.

[285] Nian Z, Zheng X, Dou Y (2021). Rapamycin pretreatment rescues the bone marrow AML cell elimination capacity of CAR-T cells. Clin Cancer Res.

[286] Dalerba P, Dylla SJ, Park IK (2007). Phenotypic characterization of human colorectal cancer stem cells. Proc Natl Acad Sci U S A.

[287] Wang G, Zhou X, Fucà G, et al. Fully human antibody V(H) domains to generate mono and bispecific CAR to target solid tumors. J Immunother Cancer. 2021;9(4):e002173. 10.1136/jitc-2020-002173 PMC8021891 33795386

[288] Zhao Z, Li Y, Liu W (2020). Engineered IL-7 receptor enhances the therapeutic effect of AXL-CAR-T cells on triple-negative breast cancer. Biomed Res Int.

[289] Banville AC, Wouters M, Oberg AL (2021). Co-expression patterns of chimeric antigen receptor (CAR)-T cell target antigens in primary and recurrent ovarian cancer. Gynecol Oncol.

[290] Tschumi BO, Dumauthioz N, Marti B (2018). CART cells are prone to Fas- and DR5-mediated cell death. J Immunother Cancer.

[291] Jiang W, Li T, Guo J (2021). Bispecific c-Met/PD-L1 CAR-T cells have enhanced therapeutic effects on hepatocellular carcinoma. Front Oncol.

[292] Chen C, Gu YM, Zhang F (2021). Construction of PD1/CD28 chimeric-switch receptor enhances anti-tumor ability of c-Met CAR-T in gastric cancer. Oncoimmunology.

[293] Mori JI, Adachi K, Sakoda Y (2021). Anti-tumor efficacy of human anti-c-met CAR-T cells against papillary renal cell carcinoma in an orthotopic model. Cancer Sci.

[294] Xu J, Wang Q, Xu H (2018). Anti-BCMA CAR-T cells for treatment of plasma cell dyscrasia: case report on POEMS syndrome and multiple myeloma. J Hematol Oncol.

[295] Zhao WH, Liu J, Wang BY (2018). A phase 1, open-label study of LCAR-B38M, a chimeric antigen receptor T cell therapy directed against B cell maturation antigen, in patients with relapsed or refractory multiple myeloma. J Hematol Oncol.

[296] Xu J, Chen LJ, Yang SS (2019). Exploratory trial of a biepitopic CAR T-targeting B cell maturation antigen in relapsed/refractory multiple myeloma. Proc Natl Acad Sci U S A.

[297] Shi D, Shi Y, Kaseb AO (2020). Chimeric antigen receptor-glypican-3 T-cell therapy for advanced hepatocellular carcinoma: results of phase I trials. Clin Cancer Res.

[298] Zah E, Nam E, Bhuvan V (2020). Systematically optimized BCMA/CS1 bispecific CAR-T cells robustly control heterogeneous multiple myeloma. Nat Commun.

[299] Sun B, Yang D, Dai H (2019). Eradication of hepatocellular carcinoma by NKG2D-based CAR-T cells. Cancer Immunol Res.

[300] Yang D, Sun B, Dai H (2019). T cells expressing NKG2D chimeric antigen receptors efficiently eliminate glioblastoma and cancer stem cells. J Immunother Cancer.

[301] Wang J, Chen S, Xiao W (2018). CAR-T cells targeting CLL-1 as an approach to treat acute myeloid leukemia. J Hematol Oncol.

[302] Tashiro H, Sauer T, Shum T (2017). Treatment of acute myeloid leukemia with T cells expressing chimeric antigen receptors directed to C-type lectin-like molecule 1. Mol Ther.

[303] Zhang C, Wang Z, Yang Z (2017). Phase I escalating-dose trial of CAR-T therapy targeting CEA(+) metastatic colorectal cancers. Mol Ther.

[304] Katz SC, Burga RA, Mccormack E (2015). Phase I hepatic immunotherapy for metastases study of intra-arterial chimeric antigen receptor-modified T-cell therapy for CEA+ liver metastases. Clin Cancer Res.

[305] Xu Y, Liu Q, Zhong M (2019). 2B4 costimulatory domain enhancing cytotoxic ability of anti-CD5 chimeric antigen receptor engineered natural killer cells against T cell malignancies. J Hematol Oncol.

[306] You F, Wang Y, Jiang L (2019). A novel CD7 chimeric antigen receptor-modified NK-92MI cell line targeting T-cell acute lymphoblastic leukemia. Am J Cancer Res.

[307] Liu E, Tong Y, Dotti G (2018). Cord blood NK cells engineered to express IL-15 and a CD19-targeted CAR show long-term persistence and potent antitumor activity. Leukemia.

[308] Gang M, Marin ND, Wong P (2020). CAR-modified memory-like NK cells exhibit potent responses to NK-resistant lymphomas. Blood.

[309] Klichinsky M, Ruella M, Shestova O (2020). Human chimeric antigen receptor macrophages for cancer immunotherapy. Nat Biotechnol.

[310] Zhang L, Tian L, Dai X (2020). Pluripotent stem cell-derived CAR-macrophage cells with antigen-dependent anti-cancer cell functions. J Hematol Oncol.

[311] Morrissey MA, Williamson AP, Steinbach AM (2018). Chimeric antigen receptors that trigger phagocytosis. Elife.

[312] Chu Y, Yahr A, Huang B (2017). Romidepsin alone or in combination with anti-CD20 chimeric antigen receptor expanded natural killer cells targeting Burkitt lymphoma in vitro and in immunodeficient mice. Oncoimmunology.

[313] Chu Y, Hochberg J, Yahr A (2015). Targeting CD20+ aggressive B-cell non-Hodgkin lymphoma by anti-CD20 CAR mRNA-modified expanded natural killer cells in vitro and in NSG mice. Cancer Immunol Res.

[314] Tang X, Yang L, Li Z (2018). First-in-man clinical trial of CAR NK-92 cells: safety test of CD33-CAR NK-92 cells in patients with relapsed and refractory acute myeloid leukemia. Am J Cancer Res.

[315] Gurney M, Stikvoort A, Nolan E, et al. CD38 knockout natural killer cells expressing an affinity optimized CD38 chimeric antigen receptor successfully target acute myeloid leukemia with reduced effector cell fratricide. Haematologica. 2020; Online ahead of print. 10.3324/haematol.2020.271908 PMC8804573 33375774

[316] Tasian SK, Kenderian SS, Shen F (2017). Optimized depletion of chimeric antigen receptor T cells in murine xenograft models of human acute myeloid leukemia. Blood.

[317] Wermke M, Kraus S, Ehninger A (2021). Proof of concept for a rapidly switchable universal CAR-T platform with UniCAR-T-CD123 in relapsed/refractory AML. Blood.

[318] Kerr DN, Zhang L, Sokol L (2019). Blastic plasmacytoid dendritic cell neoplasm. Curr Treat Options Oncol.

[319] Jiang H, Zhang W, Shang P (2014). Transfection of chimeric anti-CD138 gene enhances natural killer cell activation and killing of multiple myeloma cells. Mol Oncol.

[320] Kruschinski A, Moosmann A, Poschke I (2008). Engineering antigen-specific primary human NK cells against HER-2 positive carcinomas. Proc Natl Acad Sci U S A.

[321] Chu J, Deng Y, Benson DM (2014). CS1-specific chimeric antigen receptor (CAR)-engineered natural killer cells enhance in vitro and in vivo antitumor activity against human multiple myeloma. Leukemia.

[322] Tassev DV, Cheng M, Cheung NK (2012). Retargeting NK92 cells using an HLA-A2-restricted, EBNA3C-specific chimeric antigen receptor. Cancer Gene Ther.

[323] Genßler S, Burger MC, Zhang C (2016). Dual targeting of glioblastoma with chimeric antigen receptor-engineered natural killer cells overcomes heterogeneity of target antigen expression and enhances antitumor activity and survival. Oncoimmunology.

[324] Sahm C, Schönfeld K, Wels WS (2012). Expression of IL-15 in NK cells results in rapid enrichment and selective cytotoxicity of gene-modified effectors that carry a tumor-specific antigen receptor. Cancer Immunol Immunother.

[325] Altvater B, Landmeier S, Pscherer S (2009). 2B4 (CD244) signaling by recombinant antigen-specific chimeric receptors costimulates natural killer cell activation to leukemia and neuroblastoma cells. Clin Cancer Res.

[326] Kailayangiri S, Altvater B, Spurny C (2017). Targeting Ewing sarcoma with activated and GD2-specific chimeric antigen receptor-engineered human NK cells induces upregulation of immune-inhibitory HLA-G. Oncoimmunology.

[327] Zhang G, Liu R, Zhu X (2013). Retargeting NK-92 for anti-melanoma activity by a TCR-like single-domain antibody. Immunol Cell Biol.

[328] Ueda T, Kumagai A, Iriguchi S (2020). Non-clinical efficacy, safety and stable clinical cell processing of induced pluripotent stem cell-derived anti-glypican-3 chimeric antigen receptor-expressing natural killer/innate lymphoid cells. Cancer Sci.

[329] Han J, Chu J, Keung CW (2015). CAR-engineered NK cells targeting wild-type EGFR and EGFRvIII enhance killing of glioblastoma and patient-derived glioblastoma stem cells. Sci Rep.

[330] Schönfeld K, Sahm C, Zhang C (2015). Selective inhibition of tumor growth by clonal NK cells expressing an ErbB2/HER2-specific chimeric antigen receptor. Mol Ther.

[331] Zhang C, Burger MC, Jennewein L (2016). ErbB2/HER2-specific NK cells for targeted therapy of glioblastoma. J Natl Cancer Inst.

[332] Fei F, Rong L, Jiang N, et al. Targeting HLA-DR loss in hematologic malignancies with an inhibitory chimeric antigen receptor. Mol Ther. 2022;30(3):1215-26. 10.1016/j.ymthe.2021.11.013 PMC8899520 34801727

[333] Jan CI, Huang SW, Canoll P, et al. Targeting human leukocyte antigen G with chimeric antigen receptors of natural killer cells convert immunosuppression to ablate solid tumors. J Immunother Cancer. 2021;9(10):e003050. 10.1136/jitc-2021-003050 PMC8524382 34663641

[334] Li Y, Hermanson DL, Moriarity BS (2018). Human iPSC-derived natural killer cells engineered with chimeric antigen receptors enhance anti-tumor activity. Cell Stem Cell.

[335] Töpfer K, Cartellieri M, Michen S (2015). DAP12-based activating chimeric antigen receptor for NK cell tumor immunotherapy. J Immunol.

[336] Ramachandran I, Lowther DE, Dryer-Minnerly R (2019). Systemic and local immunity following adoptive transfer of NY-ESO-1 SPEAR T cells in synovial sarcoma. J Immunother Cancer.

[337] D'Angelo SP, Melchiori L, Merchant MS (2018). Antitumor activity associated with prolonged persistence of adoptively transferred NY-ESO-1 (c259)T cells in synovial sarcoma. Cancer Discov.

[338] Cinar O, Brzezicha B, Grunert C, et al. High-affinity T-cell receptor specific for MyD88 L265P mutation for adoptive T-cell therapy of B-cell malignancies. J Immunother Cancer. 2021;9(7):e002410. 10.1136/jitc-2021-002410 PMC8327818 34330762

[339] Postow MA, Sidlow R, Hellmann MD (2018). Immune-related adverse events associated with immune checkpoint blockade. N Engl J Med.

[340] Mognol GP, Spreafico R, Wong V (2017). Exhaustion-associated regulatory regions in CD8(+) tumor-infiltrating T cells. Proc Natl Acad Sci U S A.

[341] Wang F, Li B, Wei Y (2018). Tumor-derived exosomes induce PD1(+) macrophage population in human gastric cancer that promotes disease progression. Oncogenesis.

[342] Zhang X, Shi H, Yuan X (2018). Tumor-derived exosomes induce N2 polarization of neutrophils to promote gastric cancer cell migration. Mol Cancer.

[343] Johnson LR, Lee DY, Eacret JS (2021). The immunostimulatory RNA RN7SL1 enables CAR-T cells to enhance autonomous and endogenous immune function. Cell.

[344] Boker KO, Lemus-Diaz N, Rinaldi FR (2018). The impact of the CD9 tetraspanin on lentivirus infectivity and exosome secretion. Mol Ther.

[345] Li Z, Zhou X, Wei M (2019). In vitro and in vivo RNA inhibition by CD9-HuR functionalized exosomes encapsulated with miRNA or CRISPR/dCas9. Nano Lett.

[346] Cheng Q, Dai Z, Shi X (2021). Expanding the toolbox of exosome-based modulators of cell functions. Biomaterials.

[347] Pegram HJ, Lee JC, Hayman EG (2012). Tumor-targeted T cells modified to secrete IL-12 eradicate systemic tumors without need for prior conditioning. Blood.

[348] Mamonkin M, Rouce RH, Tashiro H (2015). A T-cell-directed chimeric antigen receptor for the selective treatment of T-cell malignancies. Blood.

[349] Hassan R, Ho M (2008). Mesothelin targeted cancer immunotherapy. Eur J Cancer.

[350] Noy R, Pollard JW (2014). Tumor-associated macrophages: from mechanisms to therapy. Immunity.

[351] Suryadevara CM, Desai R, Farber SH (2019). Preventing Lck activation in CAR T cells confers Treg resistance but requires 4-1BB signaling for them to persist and treat solid tumors in nonlymphodepleted hosts. Clin Cancer Res.

[352] Agliardi G, Liuzzi AR, Hotblack A (2021). Intratumoral IL-12 delivery empowers CAR-T cell immunotherapy in a pre-clinical model of glioblastoma. Nat Commun.

[353] Adachi K, Kano Y, Nagai T (2018). IL-7 and CCL19 expression in CAR-T cells improves immune cell infiltration and CAR-T cell survival in the tumor. Nat Biotechnol.

[354] Kuhn NF, Lopez AV, Li X (2020). CD103(+) cDC1 and endogenous CD8(+) T cells are necessary for improved CD40L-overexpressing CAR T cell antitumor function. Nat Commun.

